# Supplementary material for: A phylogeny-informed characterisation of global tetrapod traits addresses data gaps and biases
Source: PLoS Biol. 2024 Jul 11;22(7):e3002658. doi: 10.1371/journal.pbio.3002658 (PMC11239118; doi:10.1371/journal.pbio.3002658)
Supplement: S1 Fig — The y-axis indicates the Pearson correlation coefficient for continuous traits (body length and body mass, log10 transformed) and the proportion of correctly classified entries (accuracy) for binary traits (types of activity time and microhabitat). The data underlying this figure can be found in https://doi.org/10.5281/zenodo.10582069. (PDF) [file pbio.3002658.s001.pdf]

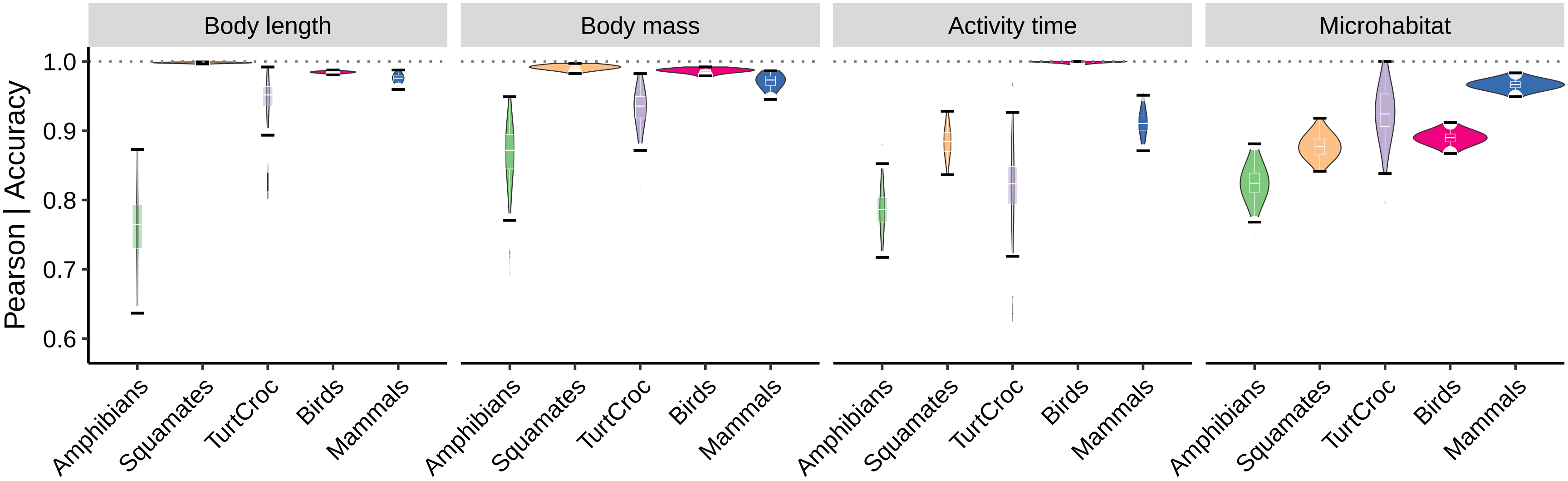
Body length
Body mass
Activity time
Microhabitat
Pearson | Accuracy
1.0
0.9
0.8
0.7
0.6
Amphibians
Squamates
TurtCroc
Birds
Mammals
