# Supplementary figures and images for: A phylogeny-informed characterisation of global tetrapod traits addresses data gaps and biases
Source: PLoS Biol. 2024 Jul 11;22(7):e3002658. doi: 10.1371/journal.pbio.3002658 (PMC11239118; doi:10.1371/journal.pbio.3002658)

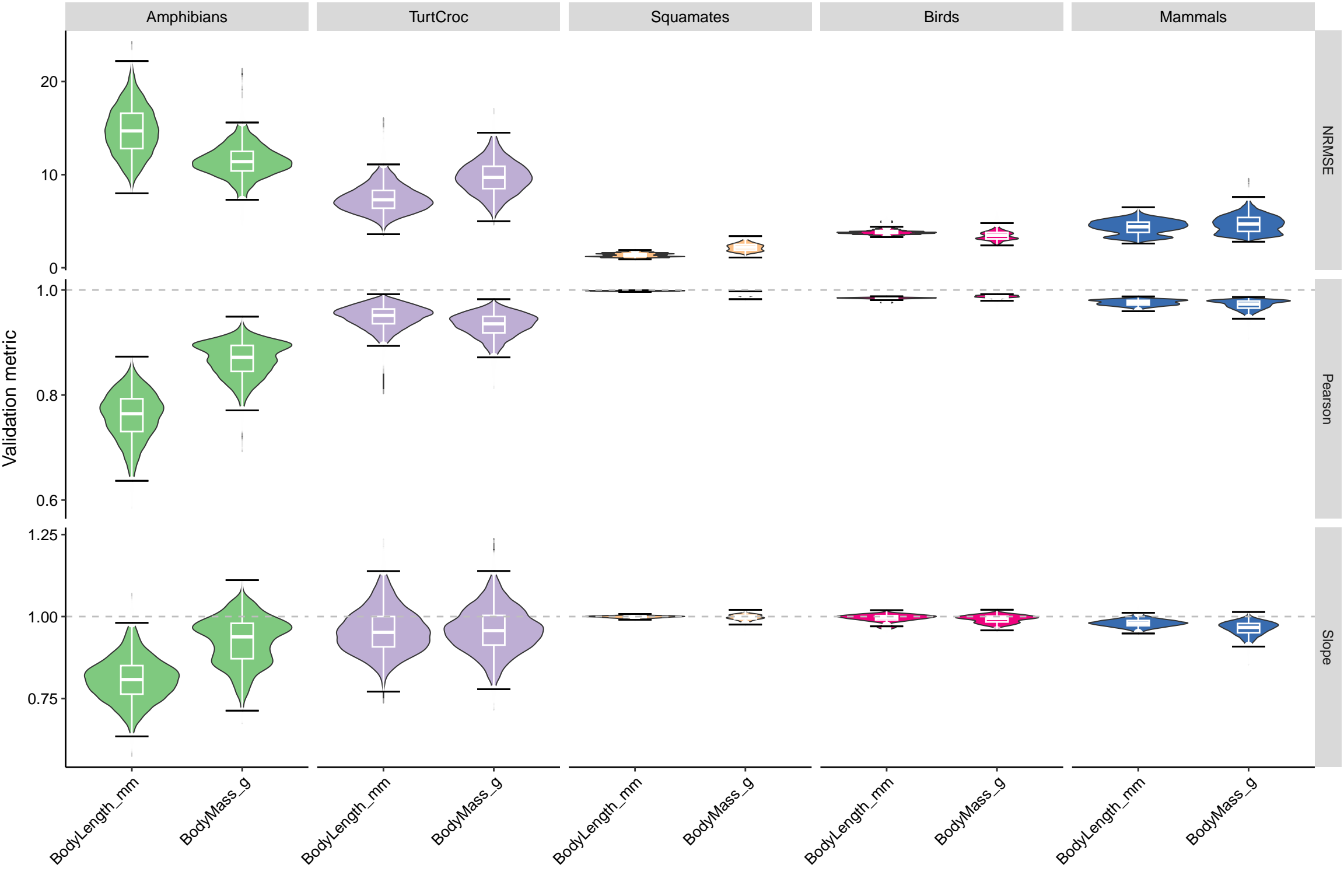

Supplement: S2 Fig — Validation metrics were computed between imputed and observed log10 attribute values, and include: NRMSE (normalised root mean square error), Pearson (Pearson correlation coefficient), and Slope (linear regression slope). The data underlying this figure can be found in https://doi.org/10.5281/zenodo.10582069. (PDF) [file pbio.3002658.s002.pdf]

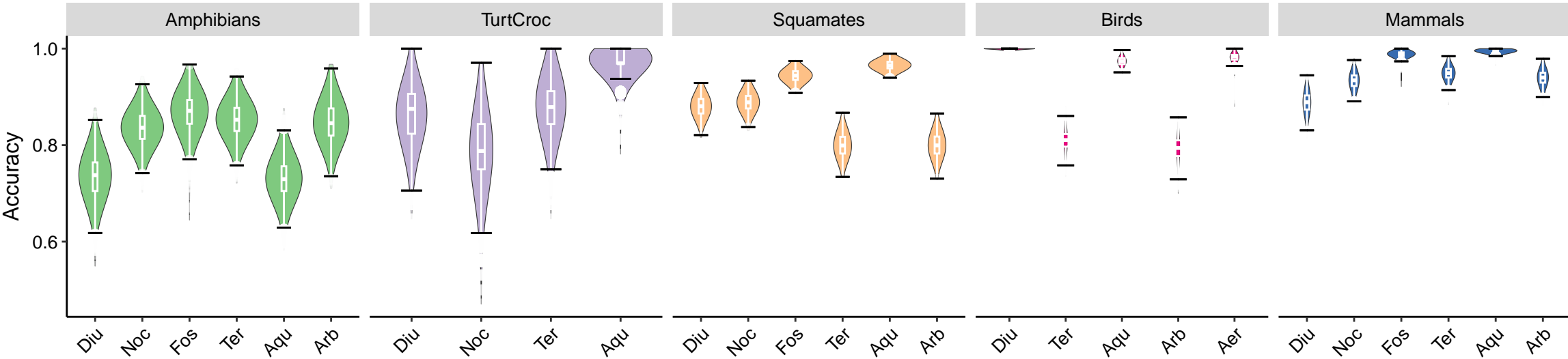

Supplement: S3 Fig — The accuracy, measured as the proportion of correctly classified entries, was computed by comparing imputed and observed binary values. Results are reported separately for different types of activity time and microhabitat. The data underlying this figure can be found in https://doi.org/10.5281/zenodo.10582069. (PDF) [file pbio.3002658.s003.pdf]

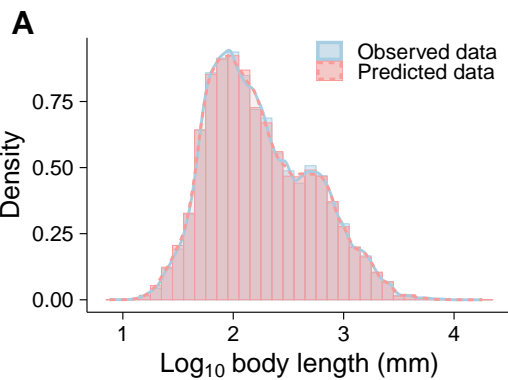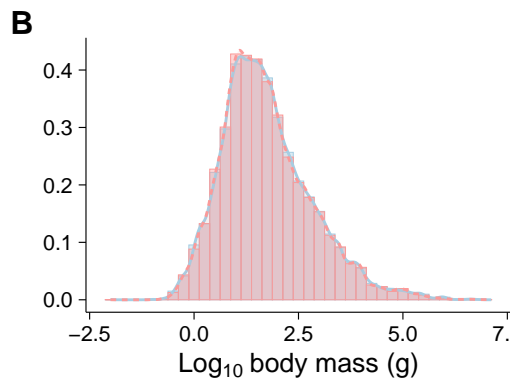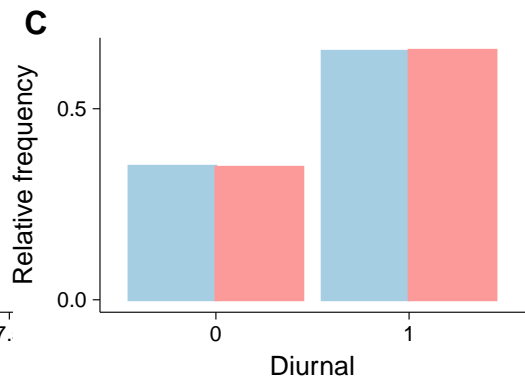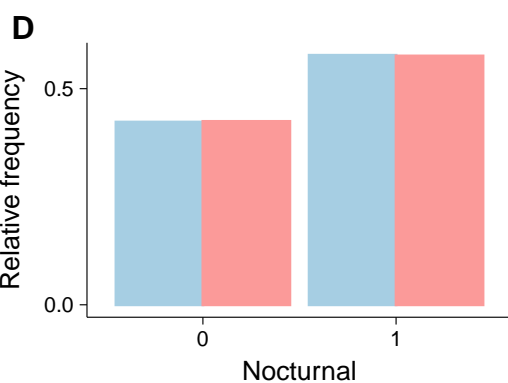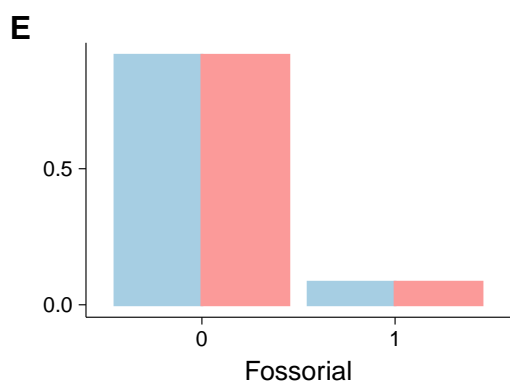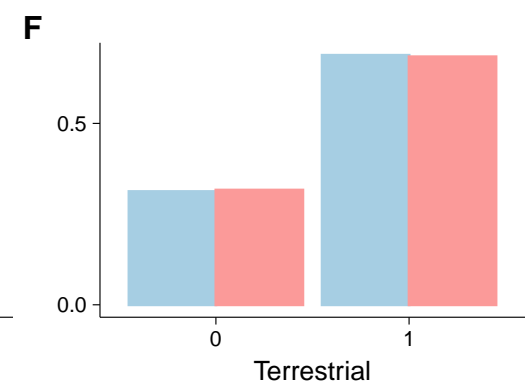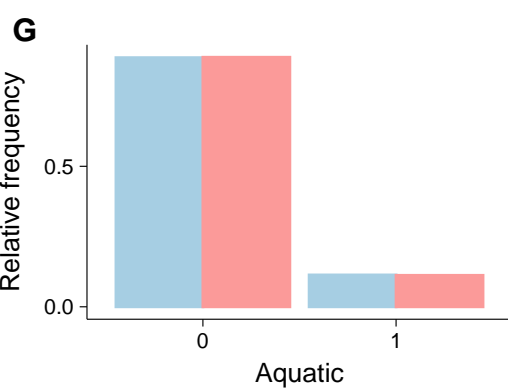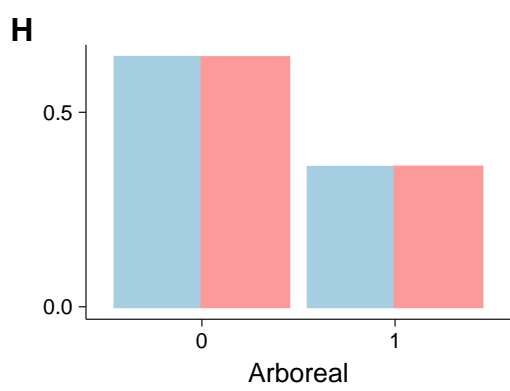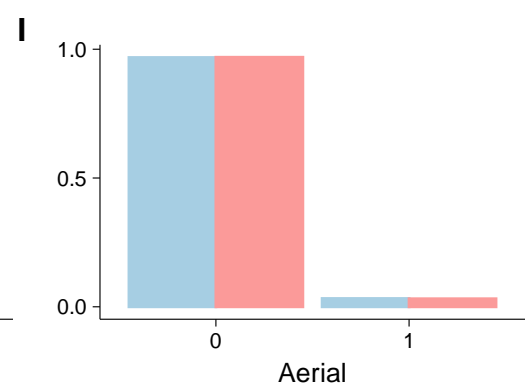

Supplement: S4 Fig — (A, B) Frequency distribution of data entry values for continuous variables (body length and body mass, log10 transformed). (C–I) Relative frequency of data entries for binary variables representing types of activity time (diurnal and nocturnal) and microhabitat (fossorial terrestrial, aquatic, arboreal, and aerial). The data underlying this figure can be found in https://doi.org/10.5281/zenodo.10582069. (PDF) [file pbio.3002658.s004.pdf]

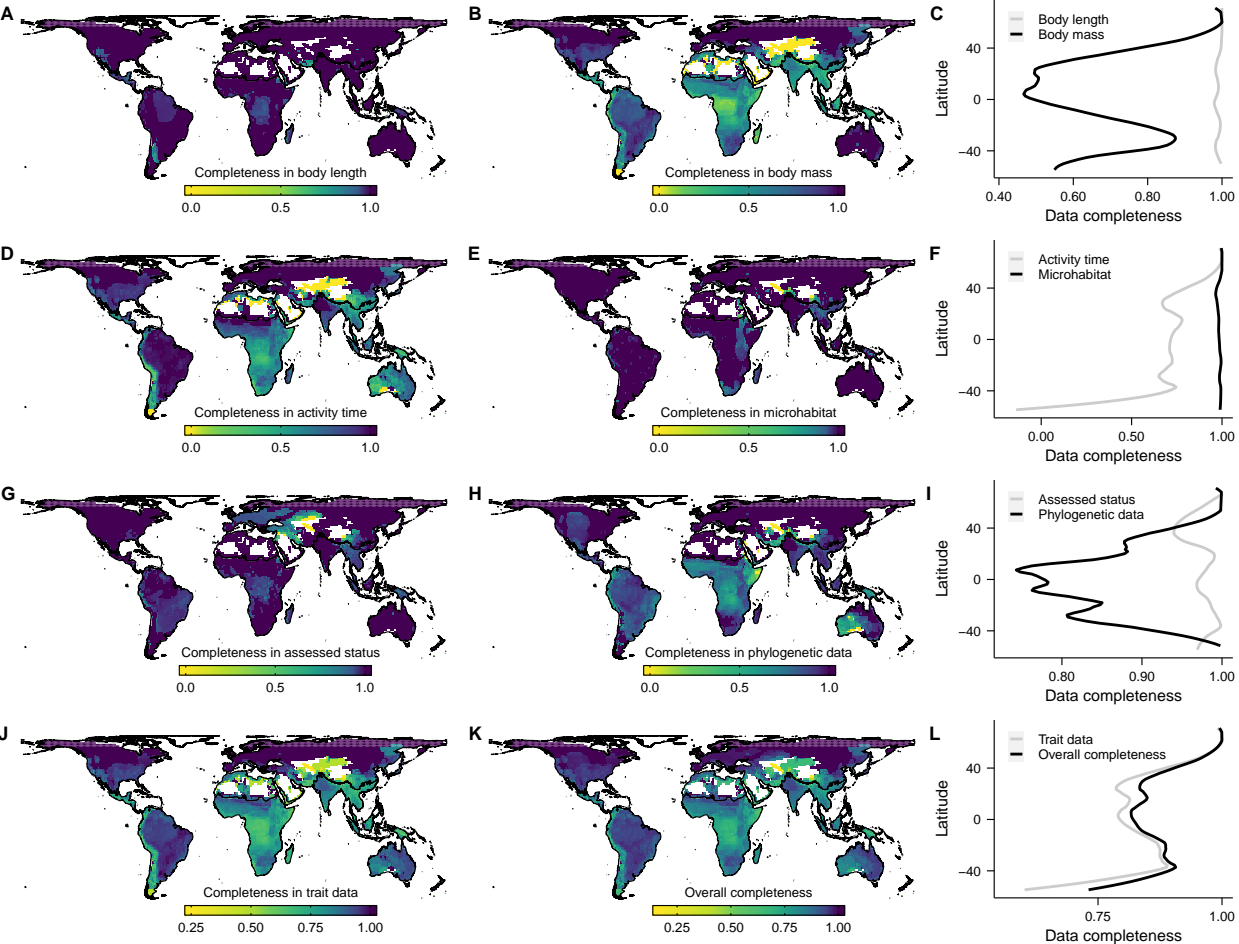

Supplement: S5 Fig — Proportion of species with observed values for: (A) body length, (B) body mass, (D) activity time, (E) microhabitat, (G) assessed threat status, (H) phylogenetic data, (J) attribute set, i.e., the average pattern for maps depicted in ABDE, and (K) the complete database, i.e., average pattern for maps depicted in ABDEGH. Maps show grid cell assemblages of 110 × 110 km size in an equal-area projection. Latitudinal plots (C, F, I, L) show the average values of these cells across latitudes. Colour ramps followed Jenks’ natural breaks classification. Boundaries of biogeographical realms were adapted from Ecoregions 2017 (https://storage.googleapis.com/teow2016/Ecoregions2017.zip). The data underlying this figure can be found in https://doi.org/10.5281/zenodo.10582069. (PDF) [file pbio.3002658.s005.pdf]

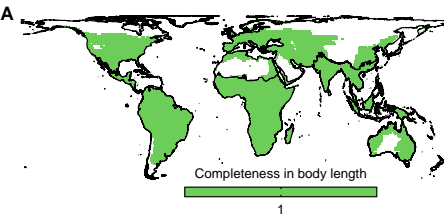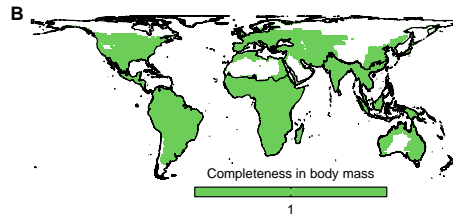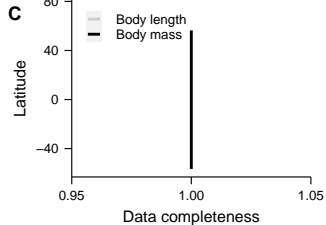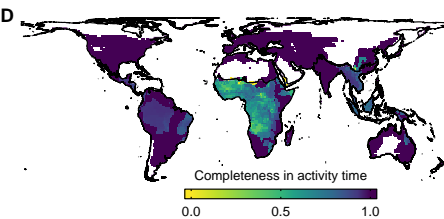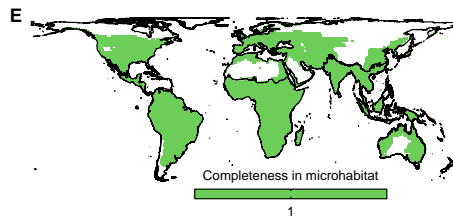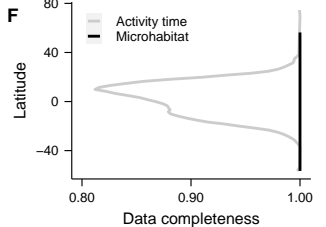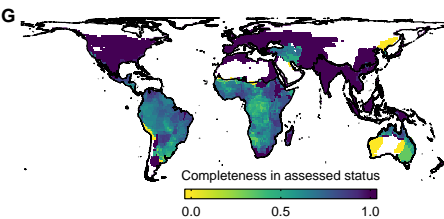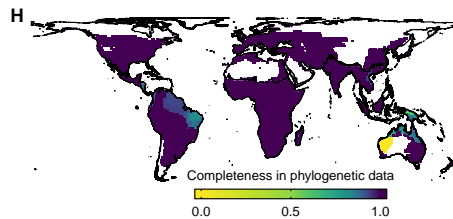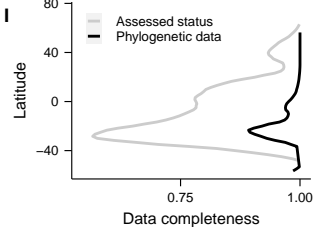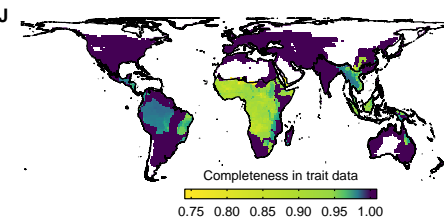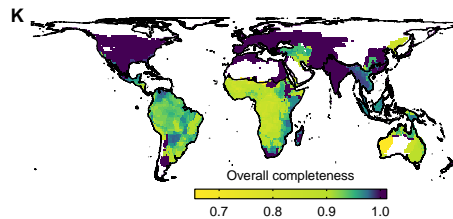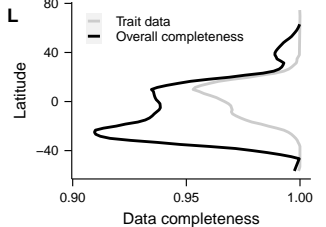

Supplement: S6 Fig — Proportion of species with observed values for: (A) body length, (B) body mass, (D) activity time, (E) microhabitat, (G) assessed threat status, (H) phylogenetic data, (J) attribute set, i.e., the average pattern for maps depicted in ABDE, and (K) the complete database, i.e., average pattern for maps depicted in ABDEGH. Maps show grid cell assemblages of 110 × 110 km size in an equal-area projection. Latitudinal plots (C, F, I, L) show the average values of these cells across latitudes. Colour ramps followed Jenks’ natural breaks classification. Boundaries of biogeographical realms were adapted from Ecoregions 2017 (https://storage.googleapis.com/teow2016/Ecoregions2017.zip). The data underlying this figure can be found in https://doi.org/10.5281/zenodo.10582069. (PDF) [file pbio.3002658.s006.pdf]

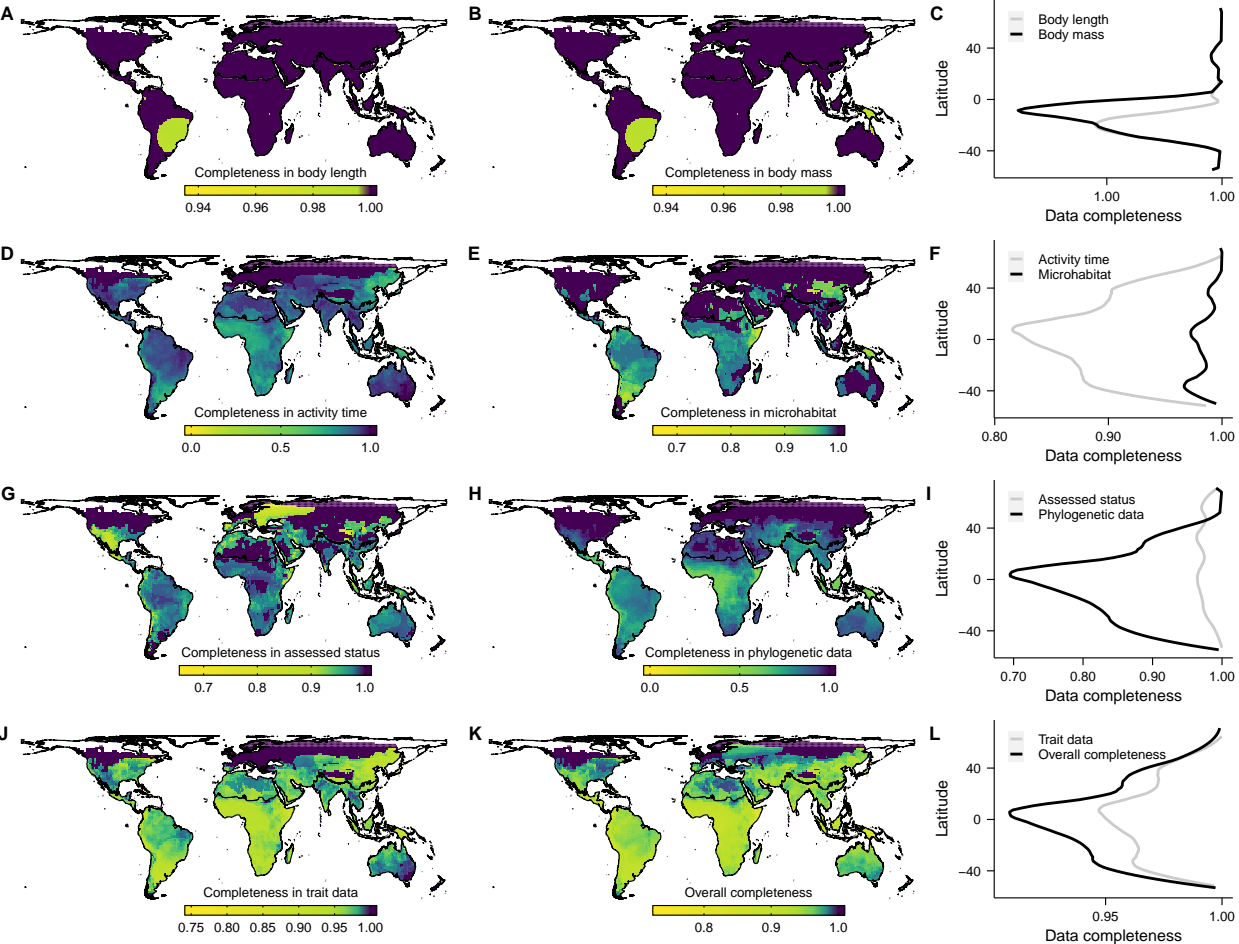

Supplement: S7 Fig — Proportion of species with observed values for: (A) body length, (B) body mass, (D) activity time, (E) microhabitat, (G) assessed threat status, (H) phylogenetic data, (J) attribute set, i.e., the average pattern for maps depicted in ABDE, and (K) the complete database, i.e., average pattern for maps depicted in ABDEGH. Maps show grid cell assemblages of 110 × 110 km size in an equal-area projection. Latitudinal plots (C, F, I, L) show the average values of these cells across latitudes. Colour ramps followed Jenks’ natural breaks classification. Boundaries of biogeographical realms were adapted from Ecoregions 2017 (https://storage.googleapis.com/teow2016/Ecoregions2017.zip). The data underlying this figure can be found in https://doi.org/10.5281/zenodo.10582069. (PDF) [file pbio.3002658.s007.pdf]

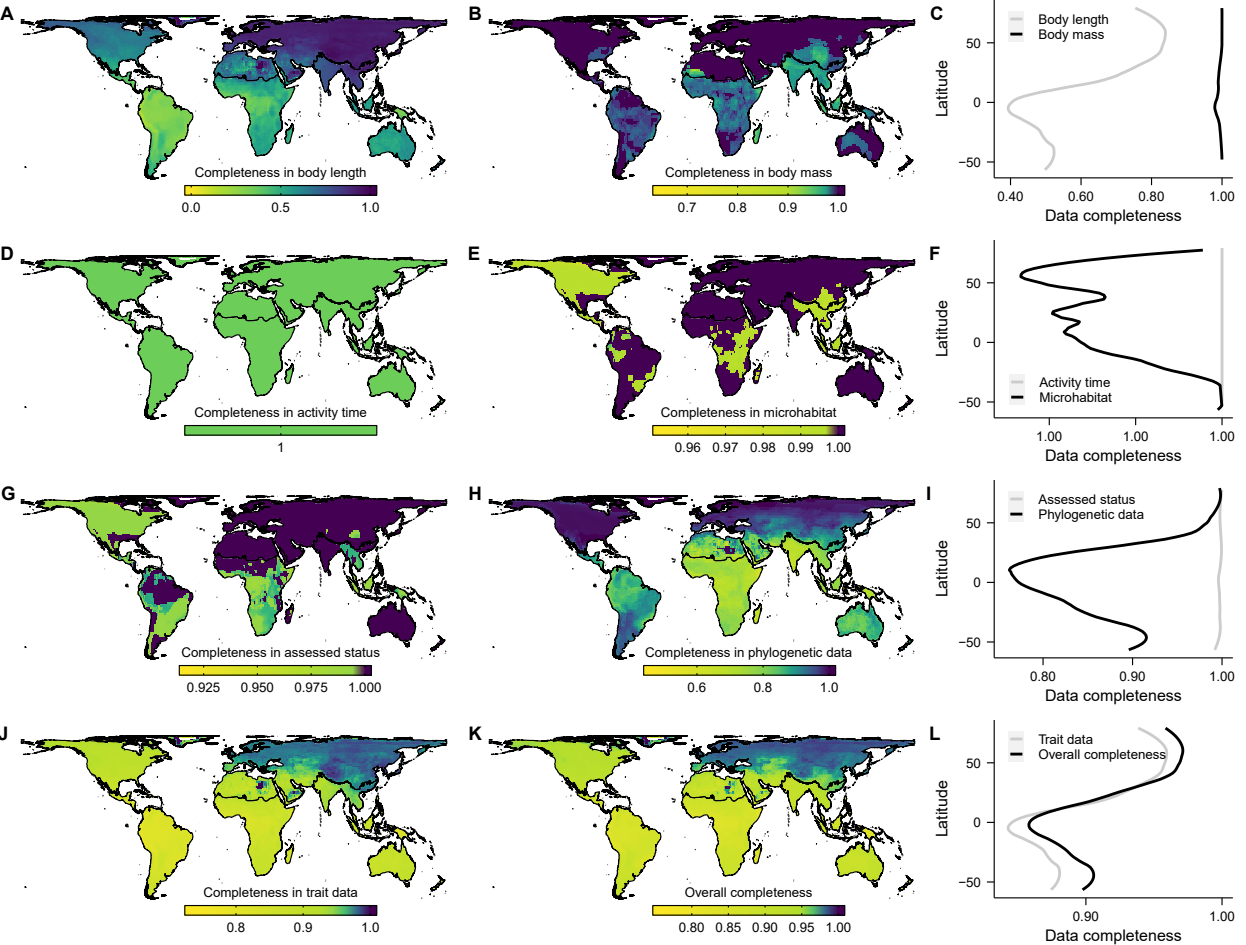

Supplement: S8 Fig — Proportion of species with observed values for: (A) body length, (B) body mass, (D) activity time, (E) microhabitat, (G) assessed threat status, (H) phylogenetic data, (J) attribute set, i.e., the average pattern for maps depicted in ABDE, and (K) the complete database, i.e., average pattern for maps depicted in ABDEGH. Maps show grid cell assemblages of 110 × 110 km size in an equal-area projection. Latitudinal plots (C, F, I, L) show the average values of these cells across latitudes. Colour ramps followed Jenks’ natural breaks classification. Boundaries of biogeographical realms were adapted from Ecoregions 2017 (https://storage.googleapis.com/teow2016/Ecoregions2017.zip). The data underlying this figure can be found in https://doi.org/10.5281/zenodo.10582069. (PDF) [file pbio.3002658.s008.pdf]

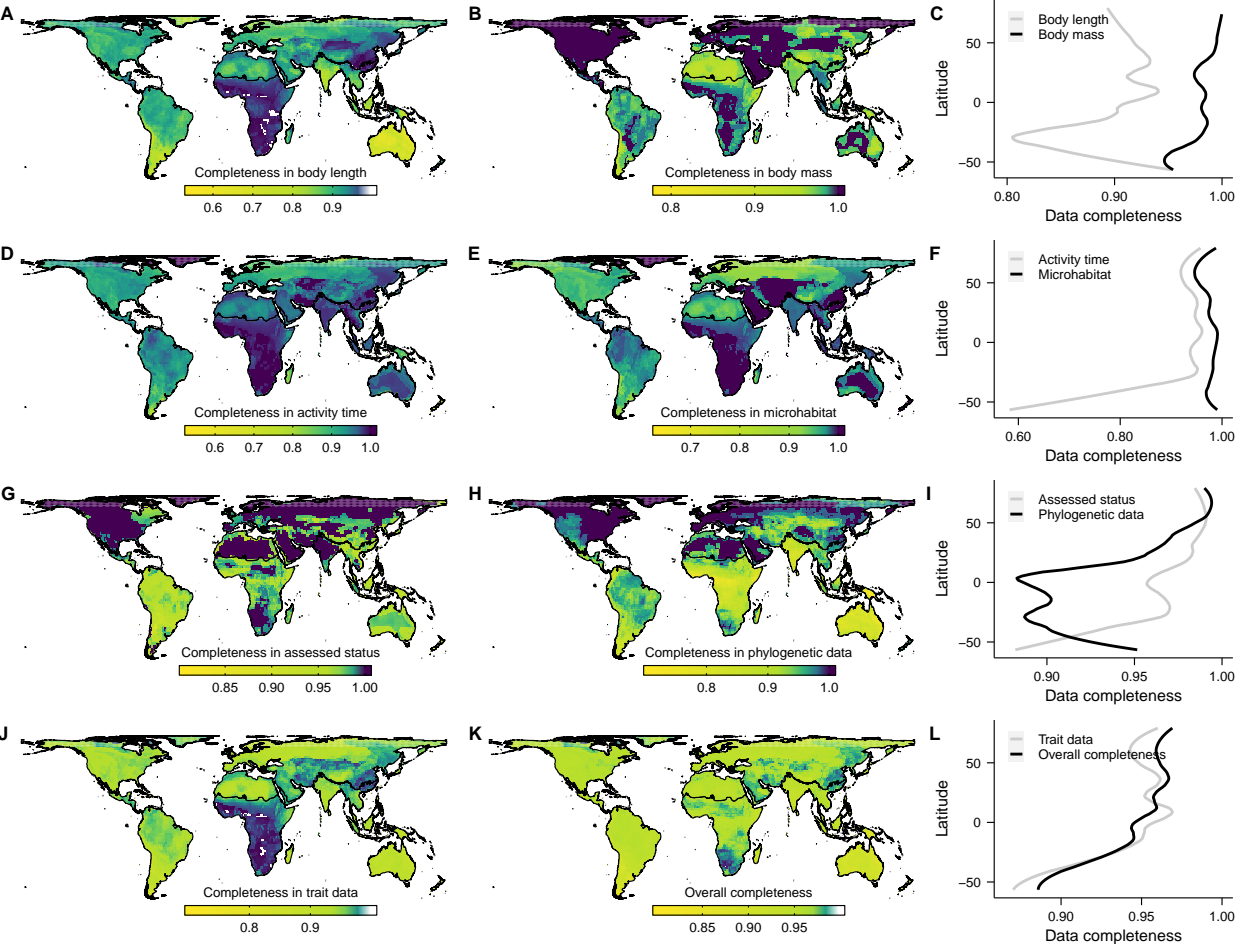

Supplement: S9 Fig — Proportion of species with observed values for: (A) body length, (B) body mass, (D) activity time, (E) microhabitat, (G) assessed threat status, (H) phylogenetic data, (J) attribute set, i.e., the average pattern for maps depicted in ABDE, and (K) the complete database, i.e., average pattern for maps depicted in ABDEGH. Maps show grid cell assemblages of 110 × 110 km size in an equal-area projection. Latitudinal plots (C, F, I, L) show the average values of these cells across latitudes. Colour ramps followed Jenks’ natural breaks classification. Boundaries of biogeographical realms were adapted from Ecoregions 2017 (https://storage.googleapis.com/teow2016/Ecoregions2017.zip). The data underlying this figure can be found in https://doi.org/10.5281/zenodo.10582069. (PDF) [file pbio.3002658.s009.pdf]

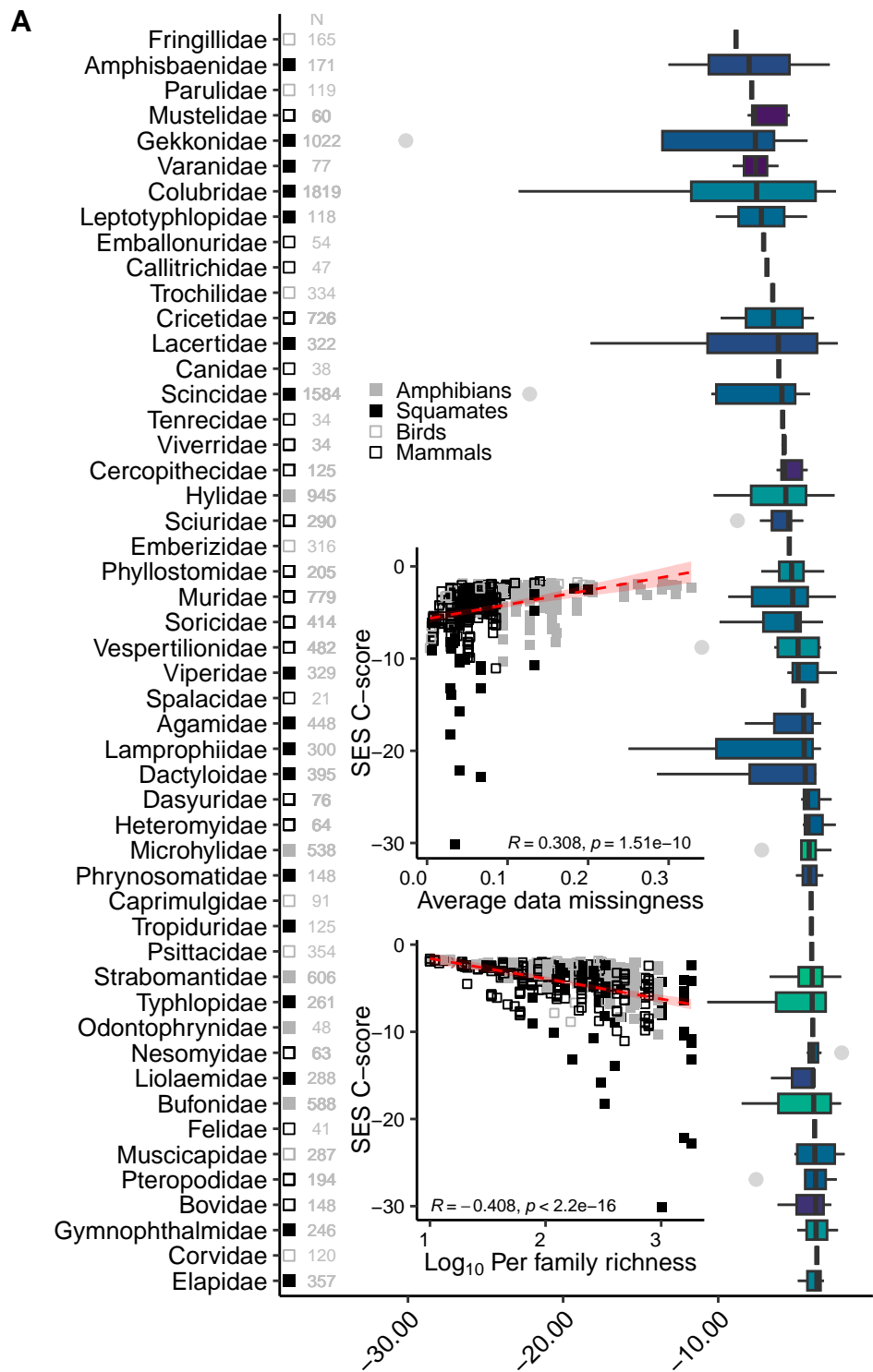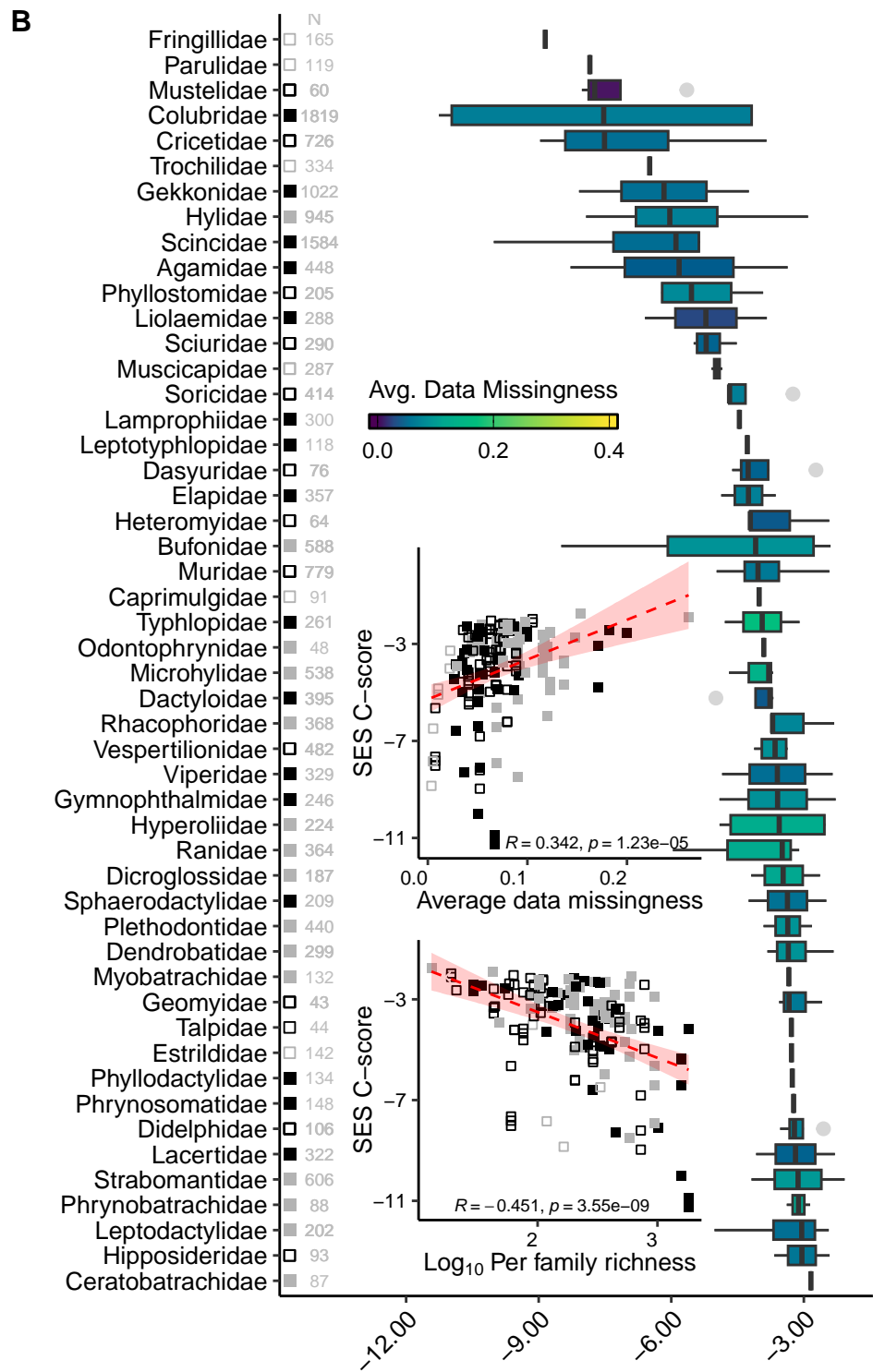

Aggregation in missing values (SES C-score)

Aggregation in missing values (SES C-score)

Supplement: S10 Fig — For each family, the aggregation metric equals the median value of the standardised effect-size (SES) of the C-score metric computed across (A) all pairwise attribute combinations or (B) attribute pairs mandatorily involving threat status. Grey numbers on the left side of each panel indicate the per-family species richness. The data underlying this figure can be found in https://doi.org/10.5281/zenodo.10582069. (PDF) [file pbio.3002658.s010.pdf]

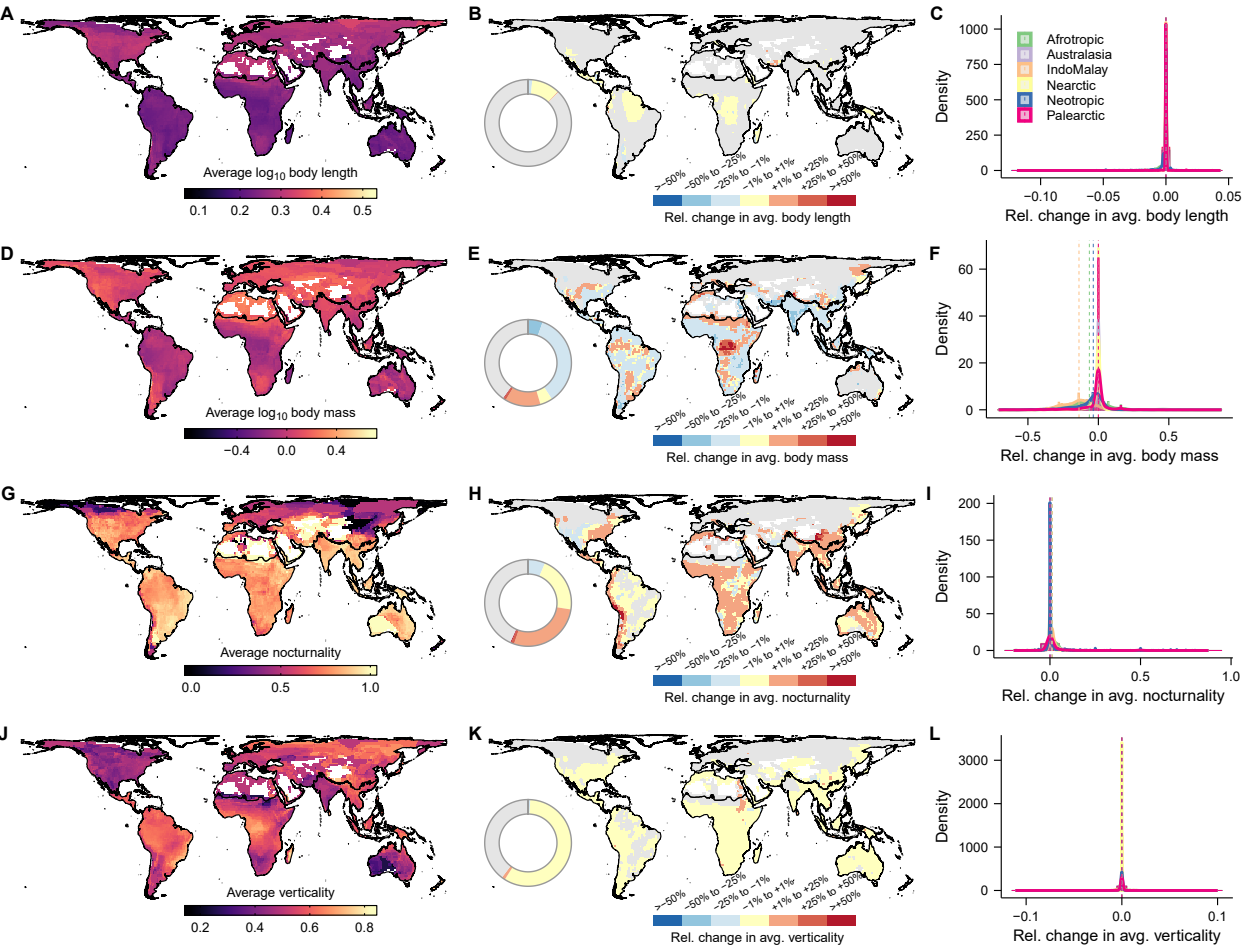

Supplement: S11 Fig — For each grid cell, maps show the average species attribute value and respective relative change in attribute value after the inclusion of imputed values for (A–C) body length, (D–F) body mass, (G–I) nocturnality, (J–L) verticality. Body length and body mass were log10 transformed before computations. Grey cells indicate assemblages without species with imputed values. Maps draw at the spatial resolution of 110 × 110 km in an equal area projection. Boundaries of biogeographical realms were adapted from Ecoregions 2017 (https://storage.googleapis.com/teow2016/Ecoregions2017.zip). The data underlying this figure can be found in https://doi.org/10.5281/zenodo.10582069. (PDF) [file pbio.3002658.s011.pdf]

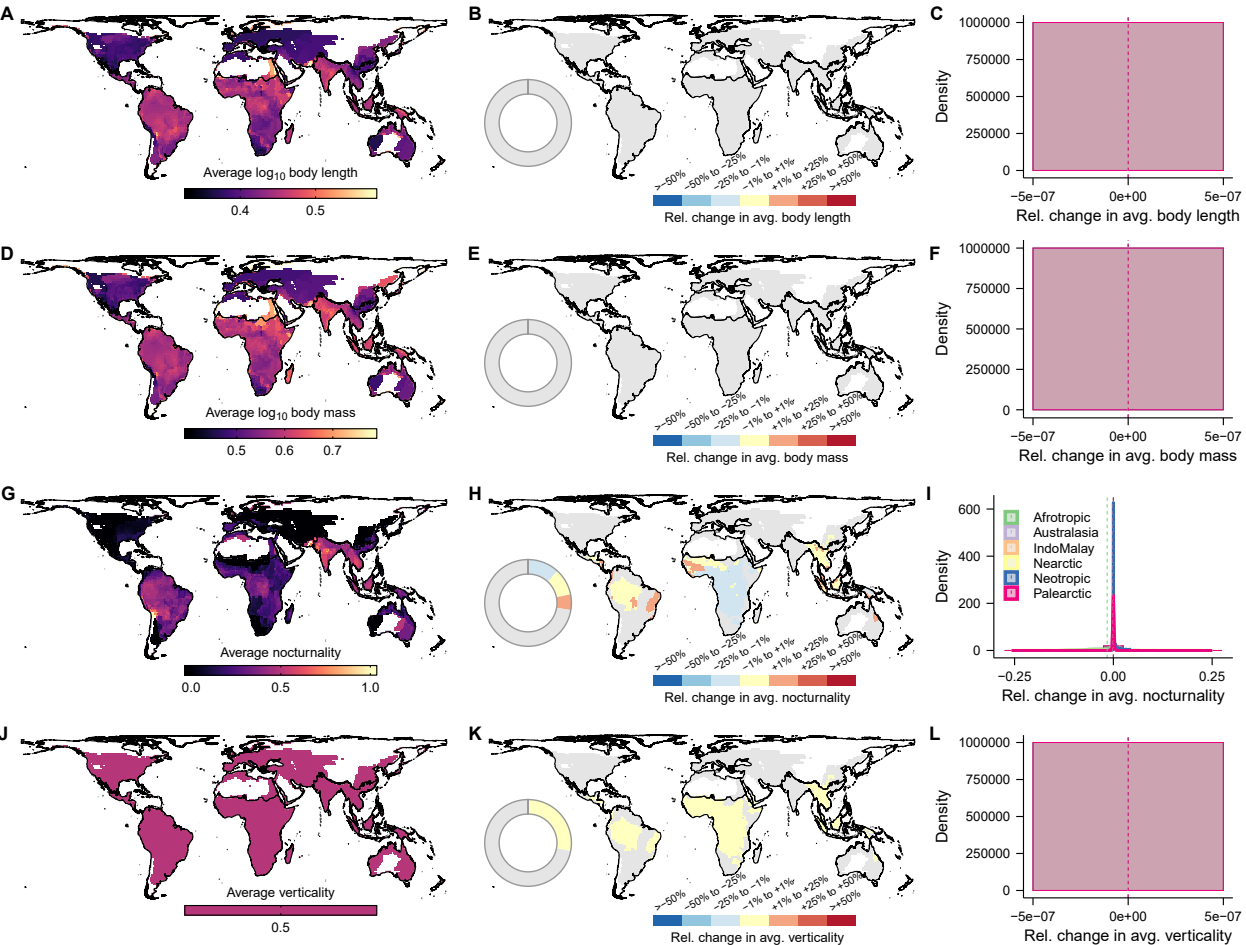

Supplement: S12 Fig — For each grid cell, maps show the average species attribute value and respective relative change in attribute value after the inclusion of imputed values for (A–C) body length, (D–F) body mass, (G–I) nocturnality, (J–L) verticality. Body length and body mass were log10 transformed before computations. Grey cells indicate assemblages without species with imputed values. Maps draw at the spatial resolution of 110 × 110 km in an equal area projection. Boundaries of biogeographical realms were adapted from Ecoregions 2017 (https://storage.googleapis.com/teow2016/Ecoregions2017.zip). The data underlying this figure can be found in https://doi.org/10.5281/zenodo.10582069. (PDF) [file pbio.3002658.s012.pdf]

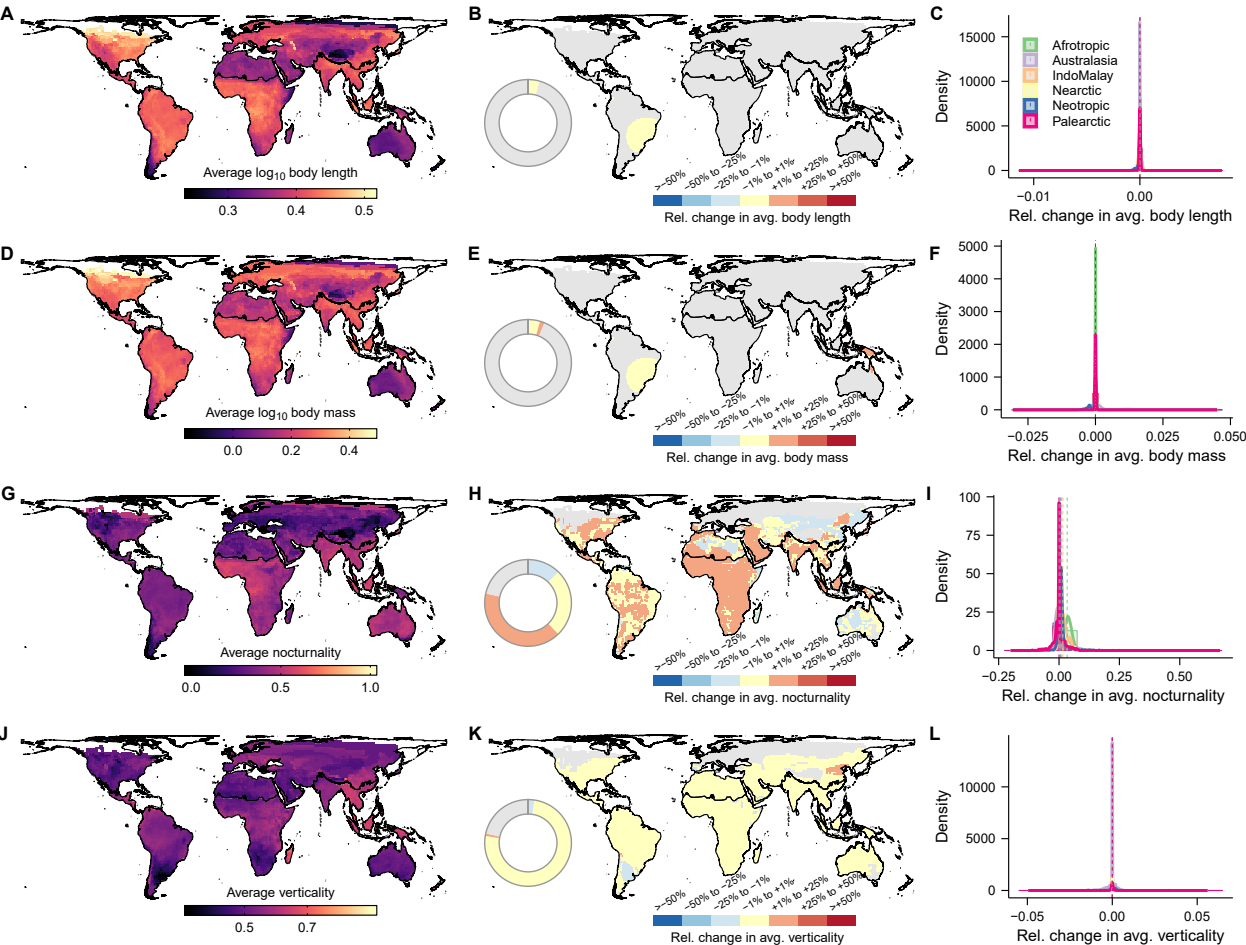

Supplement: S13 Fig — For each grid cell, maps show the average species attribute value and respective relative change in attribute value after the inclusion of imputed values for (A–C) body length, (D–F) body mass, (G–I) nocturnality, (J–L) verticality. Body length and body mass were log10 transformed before computations. Grey cells indicate assemblages without species with imputed values. Maps draw at the spatial resolution of 110 × 110 km in an equal area projection. Boundaries of biogeographical realms were adapted from Ecoregions 2017 (https://storage.googleapis.com/teow2016/Ecoregions2017.zip). The data underlying this figure can be found in https://doi.org/10.5281/zenodo.10582069. (PDF) [file pbio.3002658.s013.pdf]

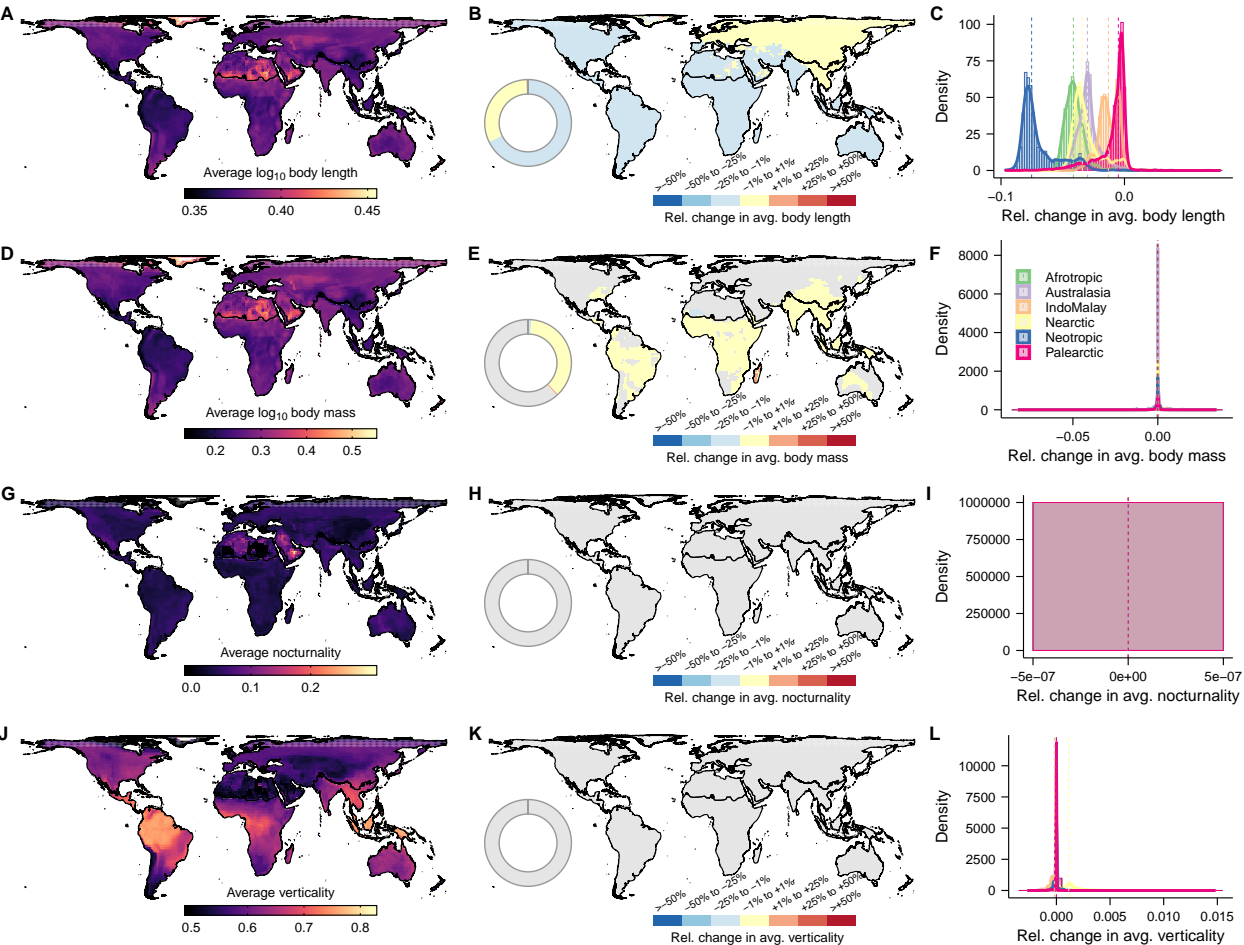

Supplement: S14 Fig — For each grid cell, maps show the average species attribute value and respective relative change in attribute value after the inclusion of imputed values for (A–C) body length, (D–F) body mass, (G–I) nocturnality, (J–L) verticality. Body length and body mass were log10 transformed before computations. Grey cells indicate assemblages without species with imputed values. Maps draw at the spatial resolution of 110 × 110 km in an equal area projection. Boundaries of biogeographical realms were adapted from Ecoregions 2017 (https://storage.googleapis.com/teow2016/Ecoregions2017.zip). The data underlying this figure can be found in https://doi.org/10.5281/zenodo.10582069. (PDF) [file pbio.3002658.s014.pdf]

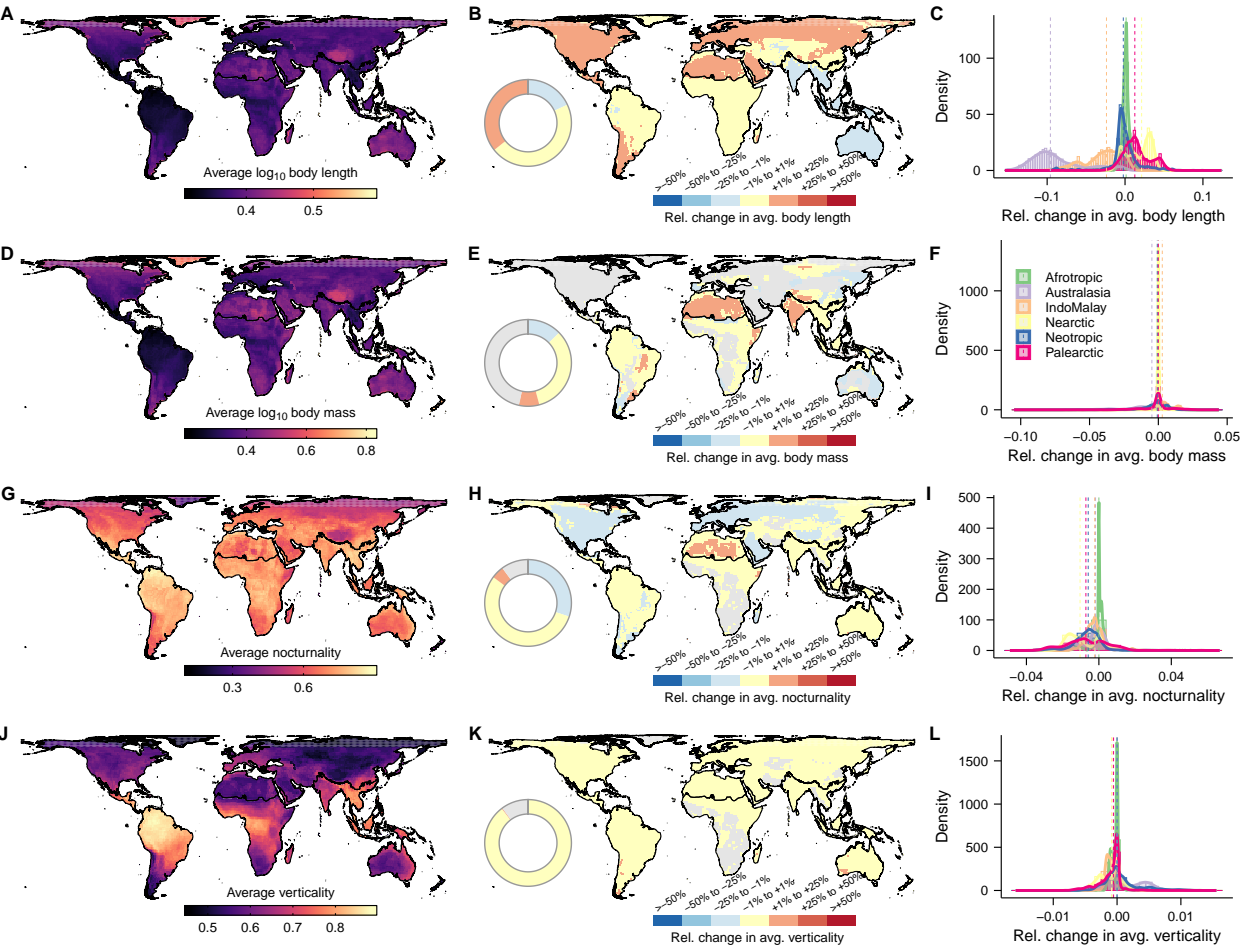

Supplement: S15 Fig — For each grid cell, maps show the average species attribute value and respective relative change in attribute value after the inclusion of imputed values for (A–C) body length, (D–F) body mass, (G–I) nocturnality, (J–L) verticality. Body length and body mass were log10 transformed before computations. Grey cells indicate assemblages without species with imputed values. Maps draw at the spatial resolution of 110 × 110 km in an equal area projection. Boundaries of biogeographical realms were adapted from Ecoregions 2017 (https://storage.googleapis.com/teow2016/Ecoregions2017.zip). The data underlying this figure can be found in https://doi.org/10.5281/zenodo.10582069. (PDF) [file pbio.3002658.s015.pdf]

Relative change in avg. trait value

- >-50%
- 50% to -25%
- 25% to -1%
- 1% to +1%
- +1% to +25%
- +25% to +50%
- >+50%
- No imputation

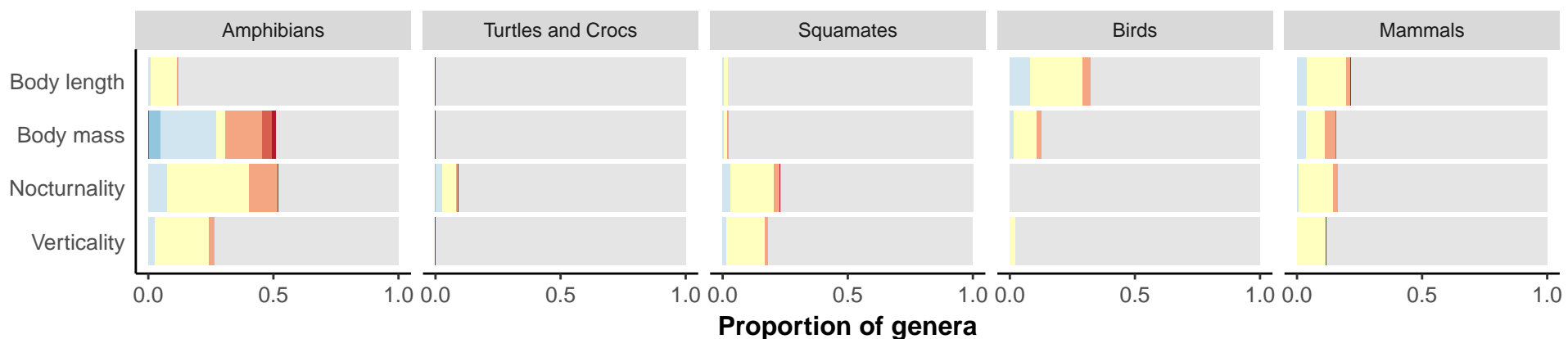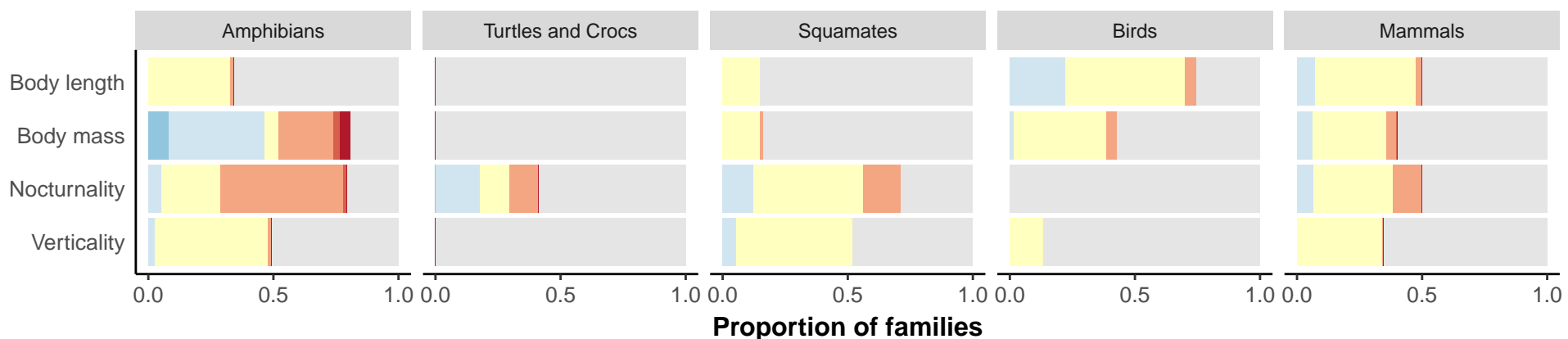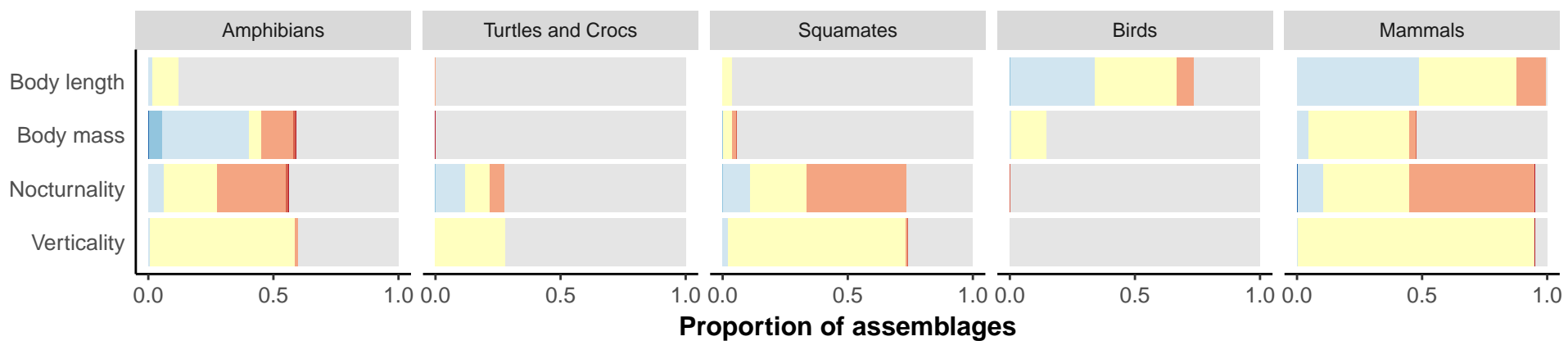

Supplement: S16 Fig — Each bar shows the relative number of genera, families, and geographical assemblages facing changes in their average species attribute value after filling missing attributes with imputed values. The data underlying this figure can be found in https://doi.org/10.5281/zenodo.10582069. (PDF) [file pbio.3002658.s016.pdf]

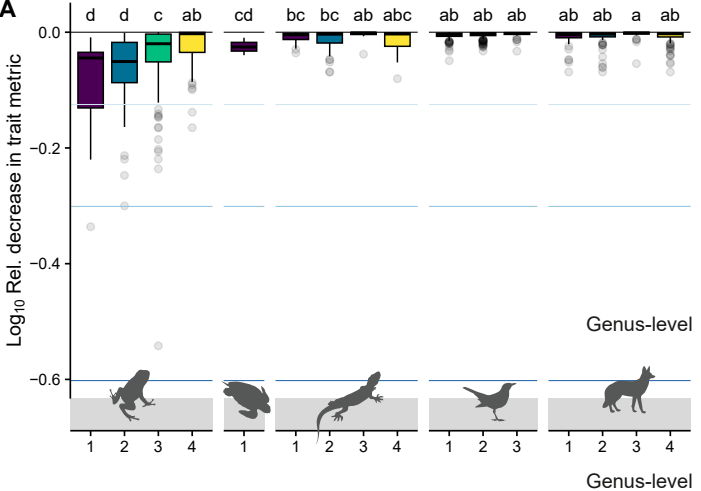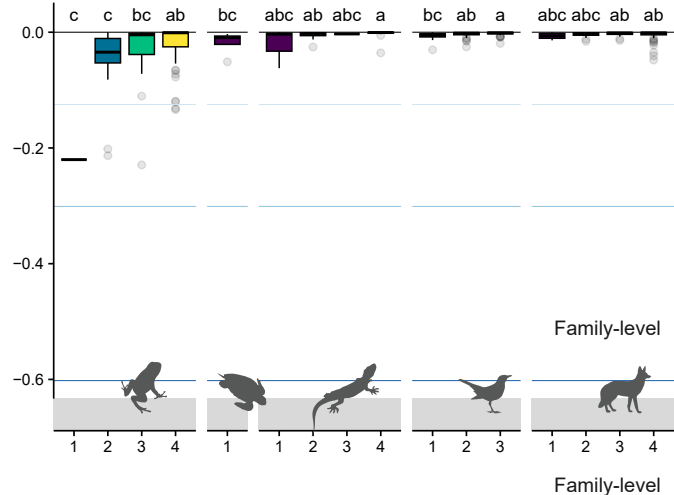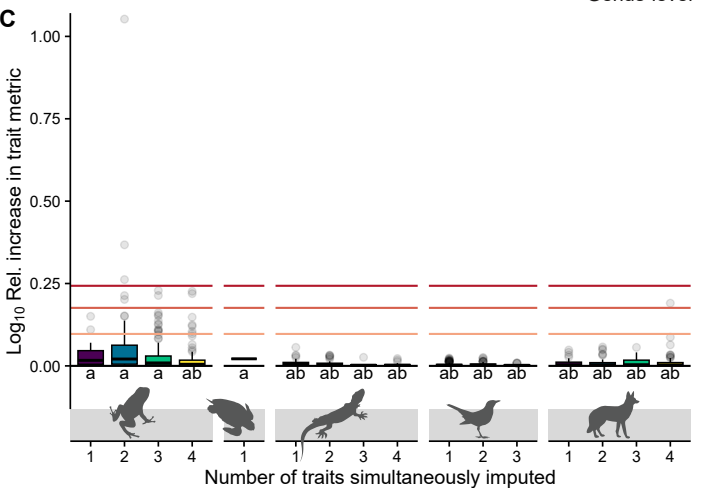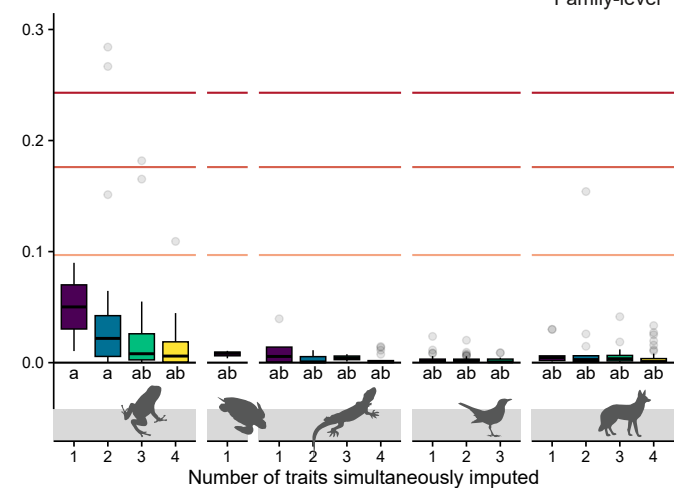

Supplement: S17 Fig — Relative decrease (A, B) or increase (C, D) in attribute metric showed at the (A, C) genus- and (B, D) family-level. Each box denotes the median (horizontal line) and the 25th and 75th percentiles. Vertical lines represent the 95% confidence intervals, and black dots are outliers. Horizontal lines denote the position of 25%, 50%, 75% of relative decrease (light to dark blue) or increase (light to dark red) in attribute metric. Small capital letters denote the results of the Kruskal–Wallis tests for the difference in medians across relative change in average attribute value. The data underlying this figure can be found in https://doi.org/10.5281/zenodo.10582069. (PDF) [file pbio.3002658.s017.pdf]

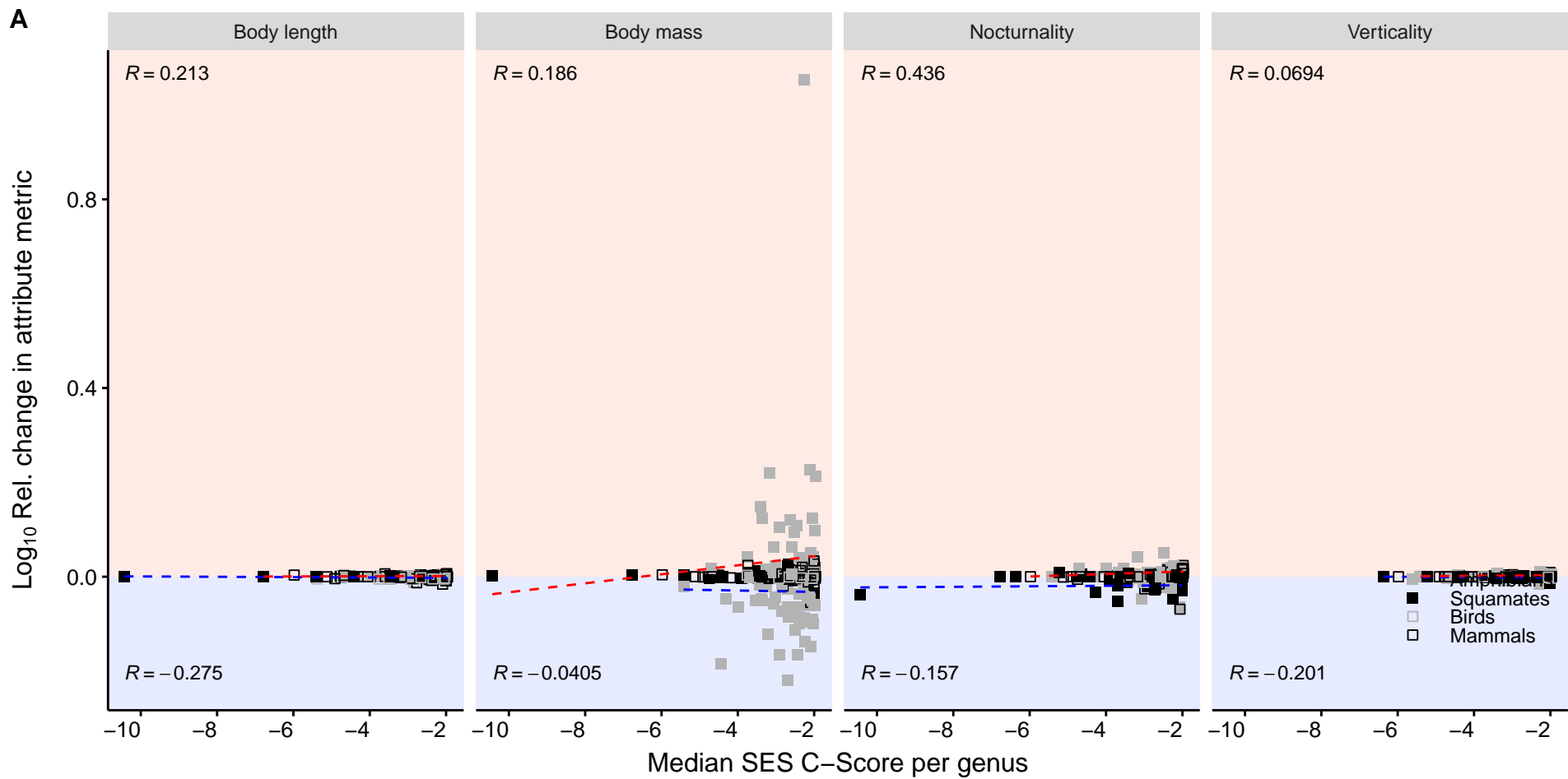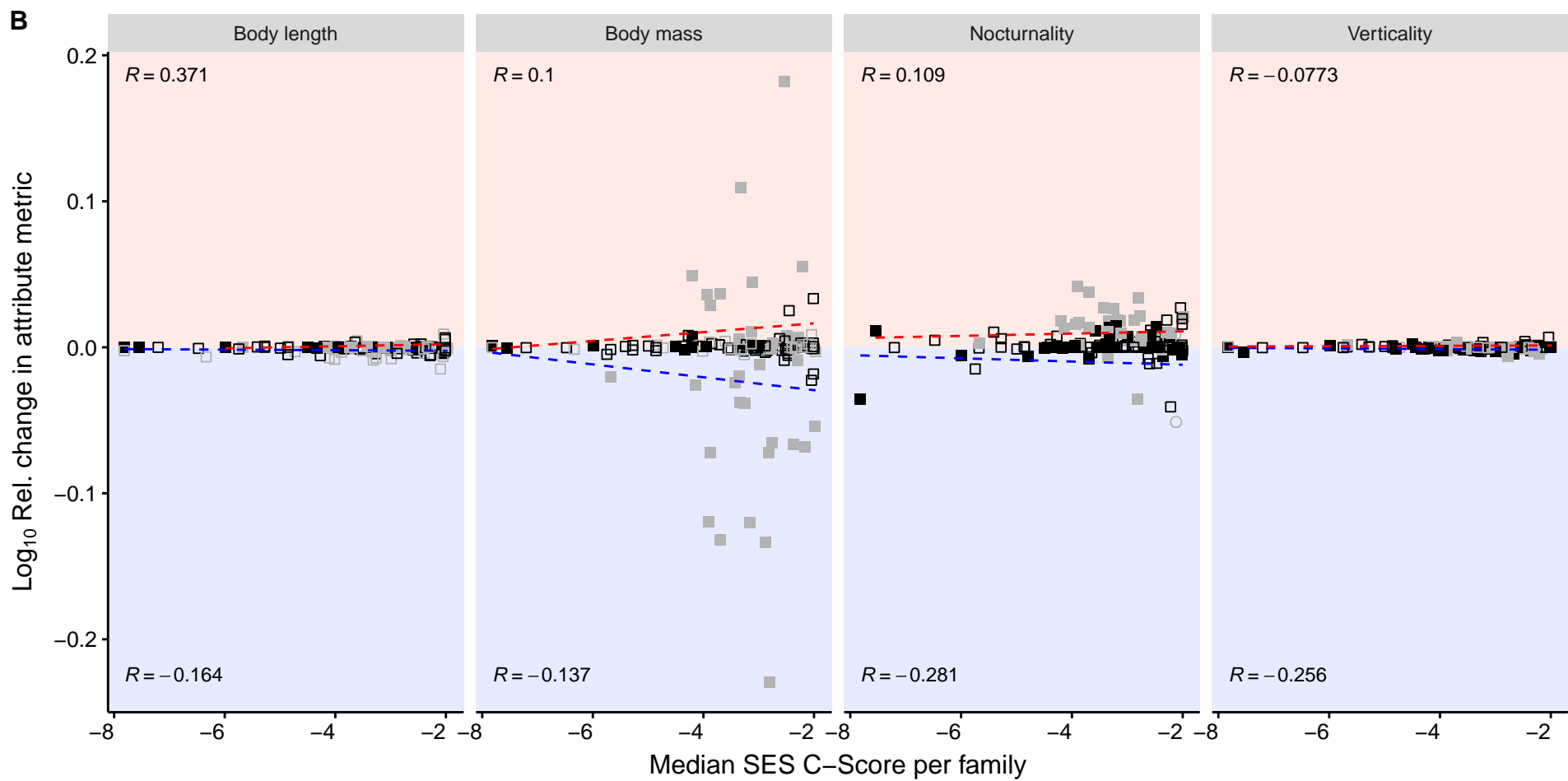

Supplement: S18 Fig — Relative changes in average attribute value per taxa (geometric mean for body length and mass, and mean for nocturnality and verticality). Each point concerns a taxonomic (A) genus or (B) family. R denotes the Spearman correlation coefficient between the relative decrease (blue) or increase (red) in the average attribute value. Only taxa with aggregated patterns of missing data were used in these plots (median Standardised Effect Size of C-score ≤ −1.96). More negative values of SES C-Score indicate a higher degree of shared missing data. The data underlying this figure can be found in https://doi.org/10.5281/zenodo.10582069. (PDF) [file pbio.3002658.s018.pdf]

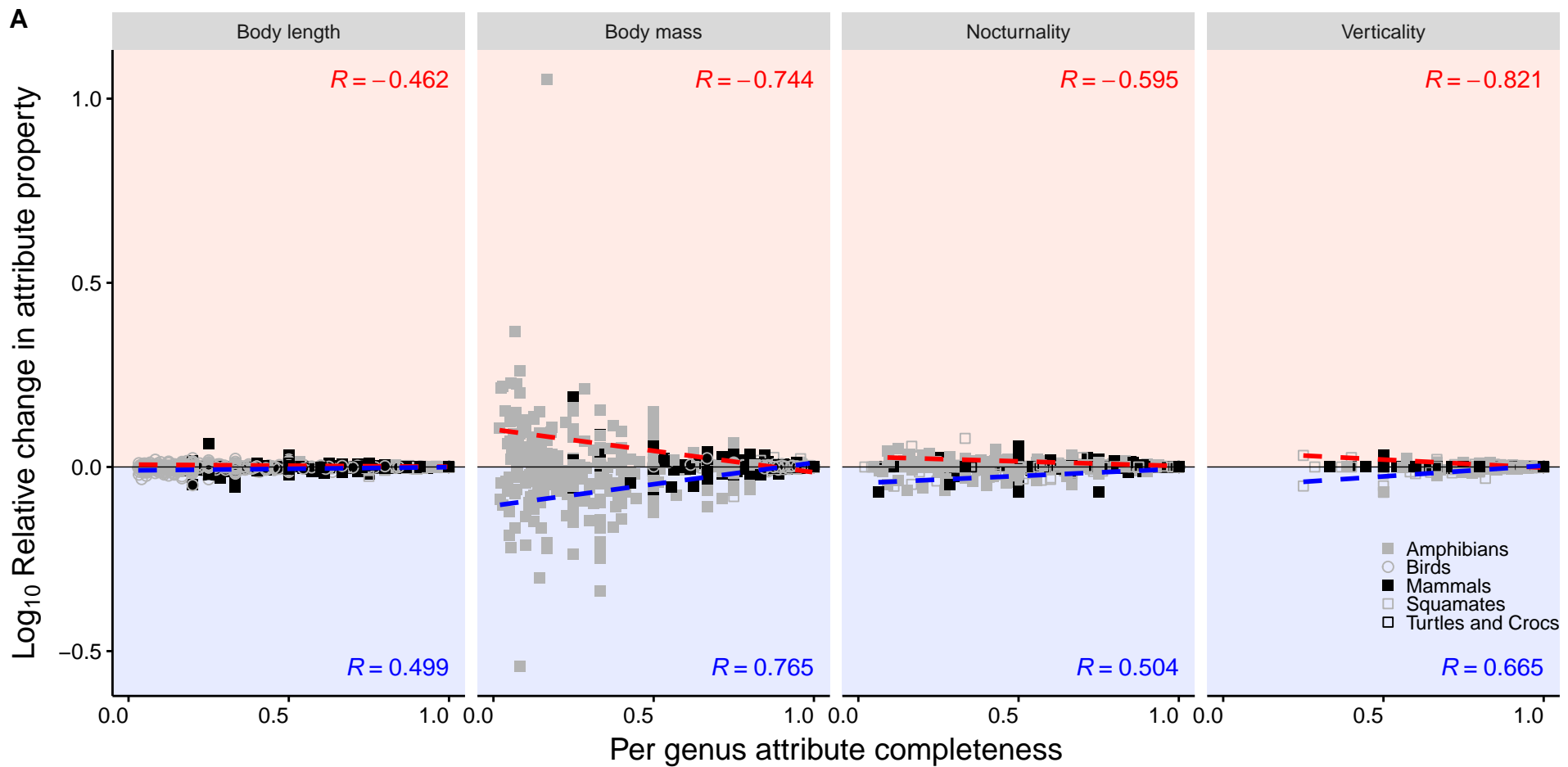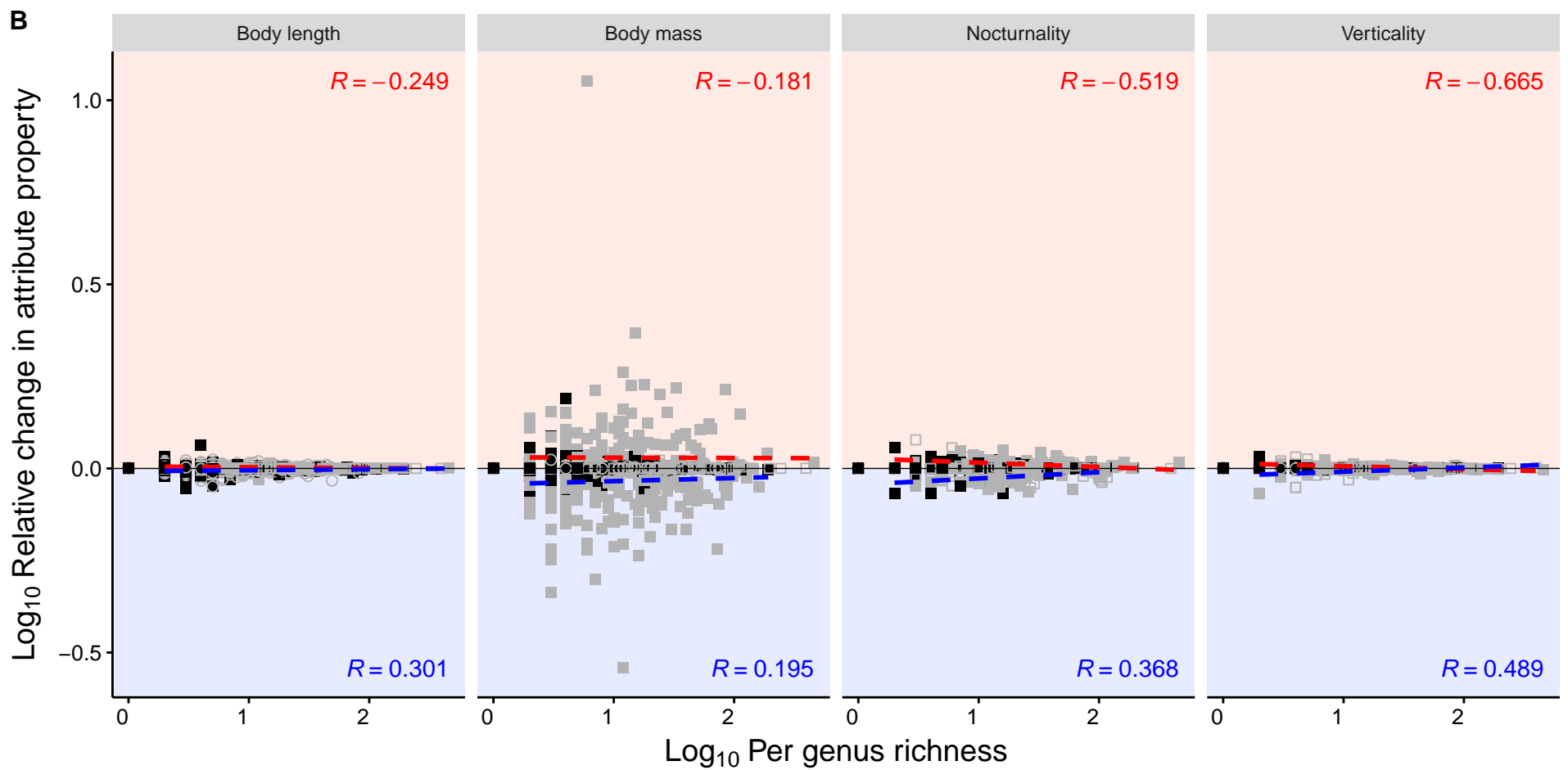

Supplement: S19 Fig — Relative changes in average attribute value per genus (geometric mean for body length and mass, and mean for nocturnality and verticality). Each point concerns a combination between the relative change in average attribute for a taxonomic genus. R denotes the Spearman correlation coefficient between the relative decrease (blue) or increase (red) in the average attribute and (A) per genus attribute completeness and (B) genus richness. The data underlying this figure can be found in https://doi.org/10.5281/zenodo.10582069. (PDF) [file pbio.3002658.s019.pdf]

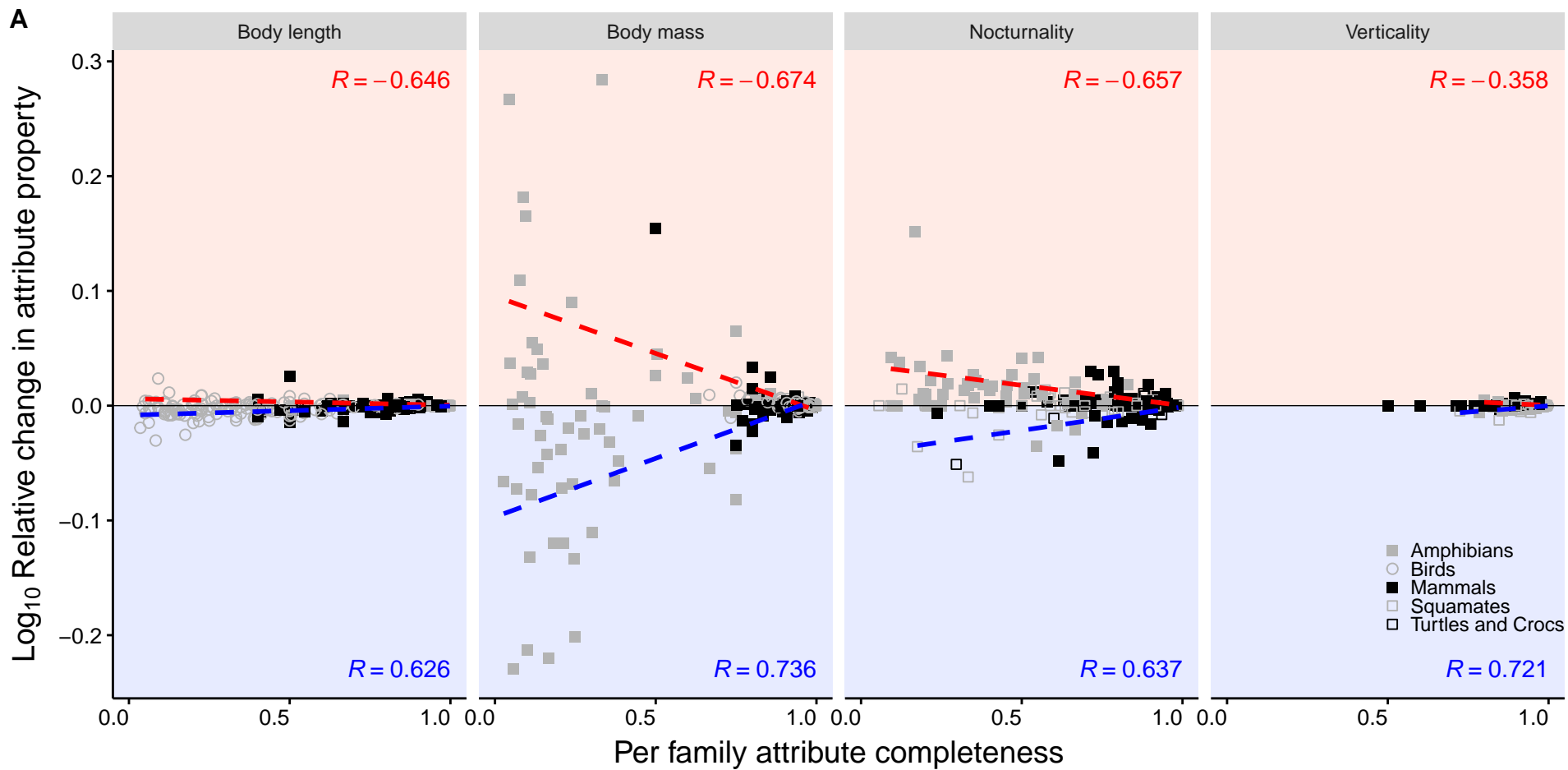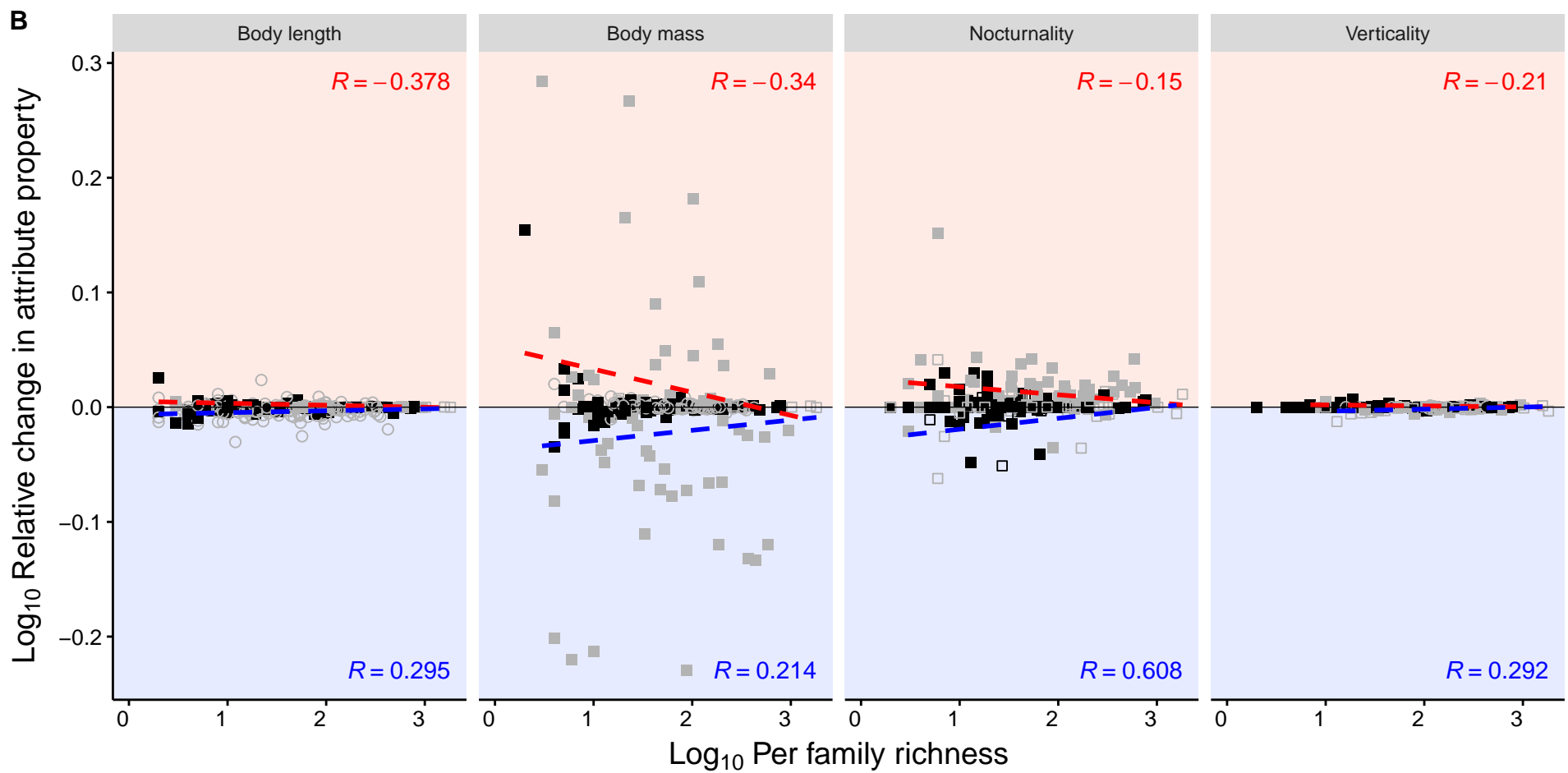

Supplement: S20 Fig — Relative changes in average attribute value per family (geometric mean for body length and mass, and mean for nocturnality and verticality). Each point concerns a combination between the relative change in average attribute for a taxonomic family. R denotes the Spearman correlation coefficient between the relative decrease (blue) or increase (red) in the average attribute and (A) per family attribute completeness and (B) family richness. The data underlying this figure can be found in https://doi.org/10.5281/zenodo.10582069. (PDF) [file pbio.3002658.s020.pdf]

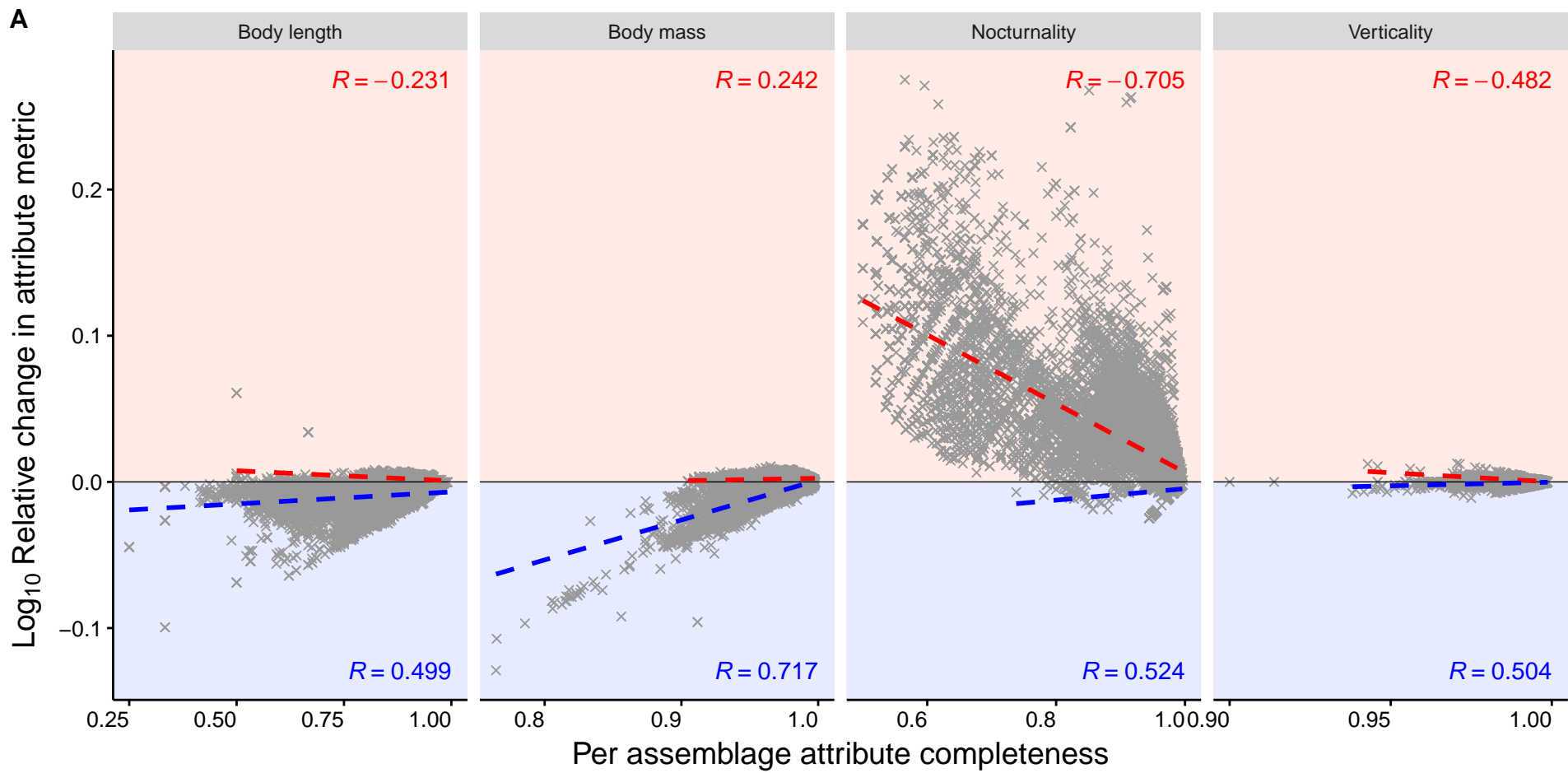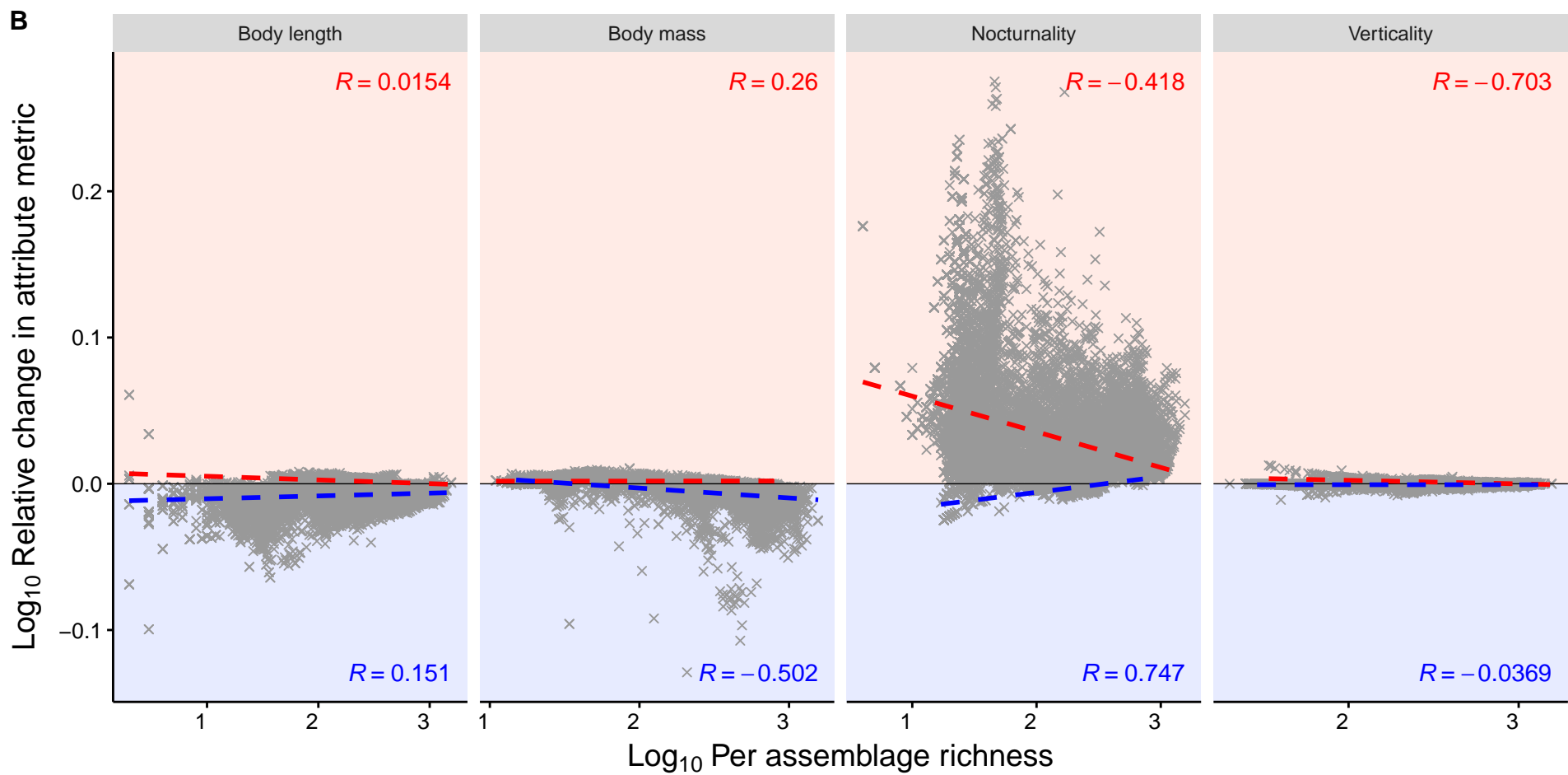

Supplement: S21 Fig — Relative changes in average attribute value per tetrapod assemblage (geometric mean for body length and mass, and mean for nocturnality and verticality). Each point concerns a combination between the relative change in average attribute for a tetrapod assemblage. R denotes the Spearman correlation coefficient between the relative decrease (blue) or increase (red) in the average attribute and (A) per assemblage attribute completeness and (B) assemblage richness. The data underlying this figure can be found in https://doi.org/10.5281/zenodo.10582069. (PDF) [file pbio.3002658.s021.pdf]
