# Supplementary material for: A phylogeny-informed characterisation of global tetrapod traits addresses data gaps and biases
Source: PLoS Biol. 2024 Jul 11;22(7):e3002658. doi: 10.1371/journal.pbio.3002658 (PMC11239118; doi:10.1371/journal.pbio.3002658)
Supplement: S1 Table — (DOCX) [file pbio.3002658.s022.docx]

**Table S1.** Variables included in the Tetrapod Traits database.

| **Category** | **Variable name** | **Description** |
| --- | --- | --- |
| Taxonomy | Scientific.Name | Binomial name in the Tetrapod tree. Categorical, 33,281 levels. |
|  | Genus | Taxonomic genus. Categorical, 5,099 levels. |
|  | Family | Taxonomic family. Categorical, 519 levels. |
|  | Suborder | Taxonomic suborder or similar higher-level taxonomic rank. Categorical, 88 levels. |
|  | Order | Taxonomic order. Categorical, 74 levels. |
|  | Class | Taxonomic class. Categorical, 4 levels. |
|  | Authority | Authority name associated with the scientific name. |
|  | YearOfDescription | Year in which the species was first described. |
| Tree | TreeTaxon | Tree-taxonomy followed. Categorical, 5 levels. |
|  | TreeImputed | Phylogenetic relationship imputed in the fully-sampled trees. Binary: 1 = Imputed, 0 = Not imputed. |
| Body Size | BodyLength_mm | Maximum body length, in millimetres (mm). |
|  | LengthMeasure | Body length measure. Categorical, 4 levels: CL = carapace length, SVL = snout-vent length, TL = total length, HBL = head-body length. |
|  | ImputedLength | Body length value imputed. Binary: 1 = Imputed, 0 = Not imputed. |
|  | SourceBodyLength | Source for body length data. |
|  | BodyMass_g | Maximum body mass in grams (g). |
|  | ImputedMass | Body mass value imputed. Binary: 1 = Imputed, 0 = Not imputed. |
|  | SourceBodyMass | Source for body mass data. |
| Activity time | Diu | Diurnal activity. Binary: 1 = Present, 0 = Absent. |
|  | Noc | Nocturnal activity. Binary: 1 = Present, 0 = Absent. |
|  | ImputedActTime | Activity Time value imputed. Binary: 1 = Imputed, 0 = Not imputed. |
|  | SourceActTime | Source for activity time data. |
|  | Nocturnality | Nocturnality score. Numeric: 0 = Diurnal, 0.5 = Cathemeral/Crepuscular, 1 = Nocturnal. |
| Microhabitat | Fos | Fossorial microhabitat use. Binary: 1 = Present, 0 = Absent. |
|  | Ter | Terrestrial microhabitat use. Binary: 1 = Present, 0 = Absent. |
|  | Aqu | Aquatic microhabitat use. Binary: 1 = Present, 0 = Absent. |
|  | Arb | Arboreal microhabitat use. Binary: 1 = Present, 0 = Absent. |
|  | Aer | Aerial microhabitat use. Binary: 1 = Present, 0 = Absent. |
|  | ImputedHabitat | Microhabitat value imputed. Binary: 1 = Imputed, 0 = Not imputed. |
|  | SourceHabitat | Source for microhabitat data. |
|  | Verticality | Verticality score. Numeric: 0 = fossorial, 0.25 = semifossorial, 0.5 = terrestrial or aquatic, 0.75 = semiarboreal, 1 = arboreal or aerial. |
| Macrohabitat | MajorHabitat_1 | Species major habitat includes Forest. Binary: 1 = Yes, 0 = No. |
|  | MajorHabitat_2 | Species major habitat includes Savanna. Binary: 1 = Yes, 0 = No. |
|  | MajorHabitat_3 | Species major habitat includes Shrubland. Binary: 1 = Yes, 0 = No. |
|  | MajorHabitat_4 | Species major habitat includes Grassland. Binary: 1 = Yes, 0 = No. |
|  | MajorHabitat_5 | Species major habitat includes Wetlands (inland). Binary: 1 = Yes, 0 = No. |
|  | MajorHabitat_6 | Species major habitat includes Rocky Areas. Binary: 1 = Yes, 0 = No. |
|  | MajorHabitat_7 | Species major habitat includes Caves & Subterranean Habitats. Binary: 1 = Yes, 0 = No. |
|  | MajorHabitat_8 | Species major habitat includes Desert. Binary: 1 = Yes, 0 = No. |
|  | MajorHabitat_9 | Species major habitat includes Marine Neritic. Binary: 1 = Yes, 0 = No. |
|  | MajorHabitat_10 | Species major habitat includes Marine Oceanic. Binary: 1 = Yes, 0 = No. |
|  | MajorHabitat_12 | Species major habitat includes Marine Intertidal. Binary: 1 = Yes, 0 = No. |
|  | MajorHabitat_13 | Species major habitat includes Marine Coastal/Supratidal. Binary: 1 = Yes, 0 = No. |
|  | MajorHabitat_14 | Species major habitat includes Artificial Terrestrial. Binary: 1 = Yes, 0 = No. |
|  | MajorHabitat_15 | Species major habitat includes Artificial Aquatic. Binary: 1 = Yes, 0 = No. |
|  | MajorHabitat_16 | Species major habitat includes Introduced Vegetation. Binary: 1 = Yes, 0 = No. |
|  | MajorHabitat_17 | Species major habitat includes Other. Binary: 1 = Yes, 0 = No. |
|  | MajorHabitatSum | Number of major habitats in which the species occur. |
|  | ImputedMajorHabitat | Major Habitat value taxonomically imputed. Binary: 1 = Imputed, 0 = Not imputed. |
|  | SourceMajorHabitat | Source for major habitat data. |
| Ecosystem | EcoTer | Species occurs in terrestrial ecosystem. Binary: 1 = Yes, 0 = No. |
|  | EcoFresh | Species occurs in freshwater ecosystem. Binary: 1 = Yes, 0 = No. |
|  | EcoMar | Species occurs in marine ecosystem. Binary: 1 = Yes, 0 = No. |
|  | EcoSystemSum | Number of ecosystems in which the species occur. |
|  | ImputedEcosystem | Ecosystem value taxonomically imputed. Binary: 1 = Imputed, 0 = Not imputed. |
|  | SourceEcosystem | Source for ecosystem data. |
| Threat Status | IUCN_Binomial | Binomial name spelling according to the IUCN red list v. 2023-1 (if available). |
|  | AssessedStatus | Assessed threat statuses from IUCN Red List. |
|  | SourceStatus | IUCN Red List version consulted for the assessed status. |
| Geography | RangeSize | Number of 110×110 km equal-area grid cells occupied by the species geographic range. |
|  | Longitude | Average within-range longitude in decimal degrees. |
|  | Latitude | Average within-range latitude in decimal degrees. |
|  | Afrotropic | Proportion of the species geographic range within the Afrotropic realm. |
|  | Australasia | Proportion of the species geographic range within the Australasia realm. |
|  | IndoMalay | Proportion of the species geographic range within the IndoMalayan realm. |
|  | Nearctic | Proportion of the species geographic range within the Nearctic realm. |
|  | Neotropic | Proportion of the species geographic range within the Neotropic realm. |
|  | Oceania | Proportion of the species geographic range within the Oceania realm. |
|  | Palearctic | Proportion of the species geographic range within the Palearctic realm. |
|  | Antarctic | Proportion of the species geographic range within the Antarctic realm. |
|  | Insularity | Species is insular endemic. Binary: 1 = Yes, 0 = No. |
|  | SourceInsularity | Source for insularity data. |
| Environment | AnnuMeanTemp | Average within-range annual mean temperature (Celsius degree). Data derived from CHELSA v. 1.2 [1]. |
|  | AnnuPrecip | Average within-range annual precipitation (mm). Data derived from CHELSA v. 1.2 [1]. |
|  | TempSeasonality | Average within-range temperature seasonality (Standard deviation × 100). Data derived from CHELSA v. 1.2 [1]. |
|  | PrecipSeasonality | Average within-range precipitation seasonality (Coefficient of Variation). Data derived from CHELSA v. 1.2 [1]. |
|  | Elevation | Average within-range elevation (metres). Data derived from topographic layers in EarthEnv [2]. |
| Human Influence | ETA50K | Average within-range estimated time to travel to cities with a population >50K in the year 2015. Data from [3]. |
|  | HumanDensity | Average within-range human population density in year 2017. Data derived from HYDE v. 3.2 [4]. |
|  | PropUrbanArea | Proportion of species range map covered by built-up area, such as towns, cities, etc. at year 2017 [4]. |
|  | PropCroplandArea | Proportion of species range map covered by cropland area, identical to FAO's category ‘Arable land and permanent crops’ at year 2017 [4]. |
|  | PropPastureArea | Proportion of species range map covered by cropland, defined as Grazing land with an aridity index > 0.5, assumed to be more intensively managed (converted in climate models) at year 2017 [4]. |
|  | PropRangelandArea | Proportion of species range map covered by rangeland, defined as Grazing land with an aridity index < 0.5, assumed to be less or not managed (not converted in climate models) at year 2017 [4]. |

1. Karger DN, Conrad O, Böhner J, Kawohl T, Kreft H, Soria-Auza RW, et al. Climatologies at high resolution for the earth’s land surface areas. Sci Data. 2017;4: 170122. doi:10.1038/sdata.2017.122

2. Amatulli G, Domisch S, Tuanmu M-N, Parmentier B, Ranipeta A, Malczyk J, et al. A suite of global, cross-scale topographic variables for environmental and biodiversity modeling. Sci Data. 2018;5: 180040. doi:10.1038/sdata.2018.40

3. Nelson A, Weiss DJ, van Etten J, Cattaneo A, McMenomy TS, Koo J. A suite of global accessibility indicators. Sci Data. 2019;6: 266. doi:10.1038/s41597-019-0265-5

4. Klein Goldewijk K, Beusen A, Doelman J, Stehfest E. Anthropogenic land use estimates for the Holocene-HYDE 3.2. Earth Syst Sci Data. 2017;9: 927–953. doi:10.5194/essd-9-927-2017
