# Supplementary material for: A phylogeny-informed characterisation of global tetrapod traits addresses data gaps and biases
Source: PLoS Biol. 2024 Jul 11;22(7):e3002658. doi: 10.1371/journal.pbio.3002658 (PMC11239118; doi:10.1371/journal.pbio.3002658)
Supplement: S2 Table — (DOCX) [file pbio.3002658.s023.docx]

**Table S2.** Number of species with natural history data available per tetrapod group.

| **Attribute/Group** | **Obs. data** | **Taxonomic Imputation** | **Phylogenetic Imputation** | **Completeness Pre-Imputing** |
| --- | --- | --- | --- | --- |
| **Amphibians (7,238 spp.)** |  |  |  |  |
| Body length^*^ | 7083 | - | 155 | 97.9% |
| Body mass^*^ | 1547 | - | 5691 | 21.4% |
| Activity time^*^ | 3501 | 183 | 3554 | 48.4% |
| Microhabitat^*^ | 6669 | 453 | 116 | 92.1% |
| Macrohabitat | 6945 | 291 | - | 95.9% |
| Ecosystem | 7092 | 146 | - | 98.0% |
| Insularity | 7238 | - | - | 100% |
| Non-DD threat status | 6388 | - | - | 88.3% |
| Expert-based range map | 7236 | - | - | >99.9% |
|  |  |  |  |  |
| **Chelonians and crocodilians (384 spp.)** |  |  |  |  |
| Body length^*^ | 384 | - | - | 100% |
| Body mass^*^ | 384 | - | - | 100% |
| Activity time^*^ | 331 | 1 | 52 | 86.2% |
| Microhabitat^*^ | 384 | - | - | 100% |
| Macrohabitat | 344 | 20 | - | 89.6% |
| Ecosystem | 384 | - | - | 100% |
| Insularity | 384 | - | - | 100% |
| Non-DD threat status | 290 | - | - | 75.5% |
| Expert-based range map | 384 | - | - | 100% |
|  |  |  |  |  |
| **Squamates (9,755 spp.)** |  |  |  |  |
| Body length^*^ | 9728 | - | 27 | 99.7% |
| Body mass^*^ | 9727 | - | 28 | 99.7% |
| Activity time^*^ | 6778 | 429 | 2548 | 69.5% |
| Microhabitat^*^ | 9162 | 333 | 260 | 93.9% |
| Macrohabitat | 9323 | 271 | - | 95.6% |
| Ecosystem | 9504 | 250 | - | 97.4% |
| Insularity | 9754 | - | - | >99.9% |
| Non-DD threat status | 8146 | - | - | 83.5% |
| Expert-based range map | 9747 | - | - | 99.9% |
|  |  |  |  |  |
| **Birds (9,993 spp)** |  |  |  |  |
| Body length^*^ | 3161 | - | 6832 | 31.6% |
| Body mass^*^ | 9436 | - | 557 | 94.4% |
| Activity time | 9993 | - | - | 100% |
| Microhabitat^*^ | 9927 | 66 | - | 99.3% |
| Macrohabitat | 9922 | 71 | - | 99.3% |
| Ecosystem | 9880 | 113 | - | 98.9% |
| Insularity | 9993 | - | - | 100% |
| Non-DD threat status | 9933 | - | - | 99.4% |
| Expert-based range map | 9993 | - | - | 100% |
|  |  |  |  |  |
| **Mammals (5,911 spp.)** |  |  |  |  |
| Body length^*^ | 4802 | - | 1109 | 81.2% |
| Body mass^*^ | 5435 | - | 476 | 91.9% |
| Activity time^*^ | 5056 | 253 | 602 | 85.5% |
| Microhabitat^*^ | 5584 | 258 | 69 | 94.5% |
| Macrohabitat | 5756 | 119 | - | 97.4% |
| Ecosystem | 5808 | 102 | - | 98.3% |
| Insularity | 5911 | - | - | 100% |
| Non-DD threat status | 5045 | - | - | 85.3% |
| Expert-based range map | 5909 | - | - | >99.9% |

^*^ Attribute with 100% of completeness post-imputation.
