# Supplementary material for: A phylogeny-informed characterisation of global tetrapod traits addresses data gaps and biases
Source: PLoS Biol. 2024 Jul 11;22(7):e3002658. doi: 10.1371/journal.pbio.3002658 (PMC11239118; doi:10.1371/journal.pbio.3002658)
Supplement: Attachment — Submitted filename: RebuttalLetter.docx [file pbio.3002658.s024.docx]

January 30, 2024

Dear Editor,

Thank you for the input on the review process and for allowing us to submit a revised version of the manuscript “*A phylogeny-informed characterization of global tetrapod traits addresses data gaps and biases*”. The earlier version of the manuscript ID PBIOLOGY-D-23-01170R1 was reviewed and all the reviewers’ suggestions and comments were carefully considered.

We hope that the current changes together with the answers we provide below adequately address all the points of concern raised by the reviewers. We also thank the reviewers for their commitment devoted to the revision of our manuscript, and for their constructive feedback on our initial submission, which significantly contributed to improve the overall quality of the article.

Overall, we reviewed and slightly expanded the occurrence database, incorporated a new imputation modelling approach (multiple phylogenetic imputation) to account for the uncertainty in imputed values, providing a new set of 10K imputed values per missing entry in our database. We completely rebuilt our database and remade all analysis and figures in the revised version. Code and illustrative examples of our modelling framework are available at [zenodo.org/doi/10.5281/zenodo.10582069](https://zenodo.org/doi/10.5281/zenodo.10582069).

Below is the point-by-point response (*in blue italics*) to the questions and suggestions provided by the reviewers (line numbers refer to the MSWord Main Text document with tracked changes, unless stated otherwise). All references cited herein can be found at the end of this letter. We hope the associate editor and reviewers will find the revised version more solid and clearer.

Sincerely,

Mario R. Moura, on behalf of all authors

Department of Animal Biology

Universidade Estadual de Campinas, Campinas, SP Brazil 13083-862

**_________________________**

**REFEREES' COMMENTS TO AUTHORS**

**Reviewer: 1**

This paper should not be published and the resulting "database" should not be released to the biology community. To do so would jeopardise the scientific efforts of those who use the database, and contribute to the reproducibility crisis in ecology and evolutionary biology. My reasons are as follows.

***Reply****: Thank you for dedicating your time to reviewing our manuscript. We highly value your thoughtful feedback and have taken your concerns into careful consideration, implementing changes accordingly. We would like to emphasise that each imputed data entry in our database is clearly labelled, facilitating easy identification by any user. Additionally, we have included the R-code and raw data essential for replicating our analysis, ensuring transparency and reproducibility in our research.*

The paper deals with missing values by imputation. Specifically, they use 2 imputation methods: Rphylopars and missForest. The whole approach to missing data and imputation is wrong. The authors should actually read the statistical literature on imputation, and in particular the work of Don Rubin, who is the leader in this field. There is no excuse for ignoring this literature. In fact, had the authors actually read the literature (and understood it!), they would not have made the fundamental mistake of this manuscript.

***Reply****: We followed the reviewer suggestion and removed the use of single-imputation methods. In the current version, we applied a multiple imputation method – mixgb* [1] *– that combines the tree-based algorithm XGBoost* [2] *with predictive mean matching (PMM)* [3]*, a multiple imputation technique.*

The fundamental problem is the generation and use of imputed values. The authors' approach to imputation is to produce single imputations to fill in the gaps in the database. This has been recognised as a very bad thing to do in the statistical literature for many years. The authors then go on to look for how "good" their imputations are, by measuring Pearson correlations and regression slopes between observed and imputed data, Normalised Root Mean Square Error (NRMSE), and the Proportion of falsely classified entries (PFC). In fact, these methods are quite beside the point, nonsensical and positively misleading.

***Reply****: The current analysis produced 10K imputed values per missing entry in our database. All imputed values are now provided so the reader can choose how to deal with imputation variability. We unfortunately missed references from literature qualifying our validation metrics (e.g., Pearson r, regression slopes, NRMSE, accuracy) as nonsensical and misleading. Thus, we kept the validation approach to assess the performance of mixgb models in imputing missing data.*

Single imputation is wrong because it invites the data analyst to treat the imputed data as "real" data, thus biasing any analyses and producing confidence intervals that are too tight and p-values that are too small. Thus, releasing this database onto the scientific community risks conclusions being too strong, and potentially wrong. What the authors SHOULD have done was to use Multiple Imputation (see the work of Rubin et al.). Multiple imputation involves imputing the missing data MULTIPLE times, ending up with SEVERAL datasets that all have imputed values, but are slightly different because the imputations are generated from some statistical model. Then analyses can be performed on each of the multiply-imputed datasets in turn. The results of analyses across datasets are then pooled using Rubin's rules (See Barnard and Rubin 1999) to account for the within-analysis and among-dataset sources of variation, resulting in wider confidence intervals and larger p-values since some of the data are simulated. Here we come to an important point: While single imputation invites the user to pretend that their simulated ("made-up") data have exactly the same status as the real data, multiple imputation allows the full use of the data that are real, and adjusting for the data that are simulated. It is easy to see why the calculation of Pearson correlations on observed versus imputed data (and the other methods) are of little importance. Ideally, multiple imputation involves drawing samples from the (approximate) posterior density of the missing values, conditional on the observed data and the imputation model. Multiple Imputation does NOT try to estimate what the "true" missing value should have been. This is a serious misapprehension in the biological literature and results from a complete disregard of the relevant statistical literature. It is not limited to the present authors, but is common throughout biology. It needs to be stopped.

What is the use of Rphylopars and missForest? The imputed values produced by Rphylopars are maximum likelihood estimates of the "true" values. However, to use them in subsequent analyses falls into the same trap of single imputation. These estimates are not data. They should not be treated as such. Rphylopars is a valid approach if the model that you are interested in can be fitted by that program. But the resulting imputed values should not be used for any subsequent analysis!!!

***Reply****: While it is true that the imputed values generated by Rphylopars are maximum likelihood estimates and not actual data, we believe in offering flexibility to users. Imputation technique aim to salvage valuable but incomplete data* [4–6]*. In our approach, imputed data entries in the database are appropriately labelled, providing users with the discretion to retain or remove them based on their analytical preferences.*

The missForest approach is more promising, as it uses random forests (a stochastic procedure) to produce predictions, so it can potentially produce different imputations on each run. But the authors of that software have tried to produce imputations that have the smallest NRMSE etc. So they too were interested in producing single imputations.

***Reply****: Thank you for your insightful comment. While we acknowledge the valid concern that imputed and observed data have distinct origins, we consider imputation methods as an opportunity to detect biases in natural history data. We implemented a multiple imputation approach that considers the use of predictive mean matching (PMM)* [3] *to draw values from neighbouring samples holding observed data, which helps to avoid the inclusion of values never observed in nature.*

Hopefully now the message is clear. Don't publish databases with single imputations. Instead, publish the database without any imputations and let the users do their own imputation. Then the onus is on the users to produce valid multiply imputed values to do their own analyses for their own reasons. Another approach, originally suggested by Rubin, is for the authors to do the multiple imputation and then to publish ALL the imputed datasets, allowing users to do their own analyses on each dataset and combine the analyses using Rubin's rules. This is a good idea since the present authors can have control over the imputation procedure and decide whether any particular procedure is best. Best in the sense that it produces unbiased estimates of regression parameters (regressing response variables on explanatory variables, not regressing imputed values on observed values). The regression estimates will have wider standard errors after pooling, which is appropriate since some of the data are imputed.

***Reply****: We have revised our approach accordingly to your suggestions, providing multiple imputed values and the observed data in an independent manner.*

References

Multiple Imputation was first proposed by Rubin, D. B. (1987). Multiple imputation for nonresponse in surveys. Wiley.

There have been many papers on the procedure since then. A good starting point is the book by Little, R. J. A., & Rubin, D. B. (2020). Statistical analysis with missing data (Third edition). Wiley.

Barnard, J., & Rubin, D. B. (1999). Miscellanea. Small-sample degrees of freedom with multiple imputation. Biometrika, 86(4), 948-955.

**_________________________**

**Reviewer: 2**

This is a review of the manuscript "A phylogeny-informed characterization of global tetrapod traits addresses data gaps and biases" submitted to PLOS Biology under the 'methods and resources' criteria. Overall the manuscript is well-written, although there are a handful of typos throughout that should be easy to fix with a proof-read.

***Reply****: Many thanks for your time and consideration in reviewing our manuscript. We truly appreciated it.*

I commend the authors for their attempts at aggregating data. However, I will admit that at this point, I'm not entirely sure what the contribution here is that the authors are making. Considering the scope of the methods and resources section of PLOS Biology as:

-- 'Methods' should report a novel method or improvements to current methodologies that significantly outperform the state-of-the-art methods or that show the potential to address, for the first time, a pressing biological question. Ideally, these Methods should be of broad interest.

-- 'Resources' should be exceptional and spur future research.

The authors do not provide any novel methods or improvements to current methodologies (in fact they use off-the-shelf methods for their imputations from the best I can tell), and therefore I assume the authors are providing a 'resource'. Which indeed, they are and I don't question that. However, I think more text is needed for both the interpretation of the resource as well as how it could spur future research. This is currently my main concern, which I elaborate on in the following points that are not necessarily in any particular order.

-	When I first read the abstract I was really impressed when I saw 75 attributes. I thought holy-moly what have the authors pulled together to make 75 attributes available for all (or nearly all) tetrapods. But after reading through the manuscript, and it took a while, only evident in Line 560 and below, I realized there are only really ~7 'attributes' (#3 through #9) at a species level. While I appreciate the term 'attributes' since the term 'trait' can be highly contested, I think the authors need to be much more clear with their terminology throughout. And I strongly recommend to not oversell what was done. At this point, I'm left wondering if this was intentional or not. But to count 19 attributes under macrohabitat is surely odd to me. It is one 'attribute' or life history trait/preference, but just happens there are 17 (I think) different categories that are measured. I realize the data is in wide format and not long format, but I don't think every column should be considered an attribute. Another glaring example is body size. Body size is one attribute, but you provide two different measurements of it. So this needs to be re-thought and casted differently.

***Reply****: We agree with the reviewer in that more clarity is welcome regarding our definition of species-level attributes. In the revised version, we reorganize the presentation of our database into 24 broad categories (see Table S1 in Supporting Information for details). Within each category, we present an independent attribute and its respective metadata, including sources and details whether data is imputed or not. We also provide an alternative nomenclature matching IUCN database, which should enhance applicability of TetrapodTraits. Importantly, our database harmonise phylogenetic, spatial, and trait data across global tetrapods, enabling new avenues of comparative research in tetrapod conservation, ecology and evolution.*

-	Returning to the scope for PLOS Biology, I'm not sure if the aggregation is exceptional or not. Let me explain. Obviously, a lot of this data are already available in different datasets, for specific groups like reptiles or birds etc. some of which the senior author of this manuscript has led or contributed to. The key sentence here in the paper is "We gathered information from several global, continental, and regional databases, and complemented the existing data from …..". I think it needs to be made quite clear just how much data were aggregated and how much were complemented. How much data were added by the authors? (Aside from the phylogenetic and taxonomic imputation). I think this is implicit a little bit in Table 1, but I'm not entirely sure.

***Reply****: We appreciate the reviewer´s comment. In the revised manuscript, we introduced a new figure (new Figure 1) illustrating what is novel in TetrapodTraits, highlighting the proportion of data entries derived from (i) major databases, (ii) additional sources compiled by us, and (iii) multiple imputation analyses performed. We provide additional details on the portion of observed, taxonomically or phylogenetically imputed data in Table S2.*

-	I think the 'data acquisition' could be restructured so that each of the ~7 traits/attributes are treated in turn, so they come much quicker than in line 560. And then go through each one at a time - this would add some structure to this section that currently seems a bit all over the place with the decisions made. It is important to be clear with the decisions made, and so I appreciated that.

***Reply****: The reviewer is correct. We have restructured the paragraphs in the Data Acquisition section to present traits in a more organized manner. The information now follows the same order of broad categories included in TetrapodTraits: taxonomy, body size, activity time, microhabitat, macrohabitat, ecosystem, geography, environmental preferences, and threat status (Table S1).*

-	I have another over-arching question which is why include the 'geographic and taxonomic and trait-related biases' in this manuscript. I think the manuscript may be better served if it simply focused on providing the 'resource'. Basically a data paper. Some analysis of the imputation would be good to show that imputation is taking place within reason (like the density histogram of observed vs imputed values overlaid). But in general, the 'expectations' in paragraph that is lines 105-119 seem so obvious to me, I'm unsure what is gained by going down this hole. And it follows that a lot of the figures, like the geographic maps of completeness and change etc. don't really add much to the manuscript in my opinion. These patterns of data bias are extremely well-known and have been published on over-and-over. So, what is being added here that isn't already known? At the very least this could certainly be substantially shortened.

***Reply****: We respectfully acknowledge the concerns raised by this particular reviewer. TetrapodTraits not only serves as a comprehensive resource but also incorporates carefully curated, expert-based maps harmonized with fully-sampled phylogenies recently published in the literature. By providing traits, phylogeny, and spatial data, we aim to stimulate questions in comparative research and spatial ecology.*

*Moreover, our analysis goes beyond the mapping of data bias. It involves assessing patterns of shared data missingness and illustrating the challenges in filling these gaps with additional field efforts. We demonstrate how imputation-based techniques can reveal bias directions, shedding light on which characteristics are missing the most and where geographically.*

-	I have questions/concerns about some of the traits themselves, for example, like using the 110km x 110km that Map of Life uses, and has been questioned before, but I don't think the manuscript is ready for scrutiny of this level yet.

***Reply****: Map of Life uses multiple sources of range maps (https://mol.org/datasets/?dt=range) depending on the taxonomic group. For this manuscript, Map of Life has matched authoritative expert range maps for each of the tetrapod groups* [7–9] *with the corresponding phylogenies* [9–13] *. When matching both datasets we edited the ranges of the species as needed except when species ranges matched directly with the species name in the phylogeny and no edit was needed. Edits include i) synonyms, where species ranges were direct synonyms to names in the phylogeny and ranges only needed a name change, ii) no range, where extinct and domestic species in the phylogeny did not need a range map, iii) split, where species ranges needed to be clipped from a parent species, or when parent species ranges needed part of their range removed, iv) lumps, where species ranges needed to be lumped back with another species range, v) new species, where there was no available range map and ranges were hand drawn from other literature sources or a minimum convex polygon with a buffer needed to be drawn around type and paratype localities, vi) de Novo species whose ranges needed to be removed because they are not in the phylogeny. These edits were incorporated in new datasets for each taxonomic group (i.e., phylomaps). To minimise errors related to the use of ranges maps, each species in these new datasets, i.e., phylomaps, was intersected with 110 × 110 km equal-area grid cells.*

-	If you stick with the current introduction, it would be good to couch the missingness in missing data theory and discuss the randomness in more detail - missing at random or missing completely at random. Both can (and do) arise in biodiversity datasets. See here for more background: https://link.springer.com/article/10.1007/s00265-010-1044-7

***Reply****: We have incorporated a new paragraph in the introduction to delve into the three common types of missing mechanisms found in biodiversity data: Missing Completely at Random (MCAR), Missing at Random (MAR), and Missing Not at Random (MNAR). We have also restructured the 3^rd^ paragraph of Introduction to emphasize the connection between MNAR and shared missingness in the trait data. This adjustment aims to elucidate how the underlying causes of MNAR can lead to co-missingness across multiple variables.*

-	It isn't currently clear how these data will 'spur on new research' - a lot of macroecology is already being done at large scales and using a lot of tetrapods, so what advantages of pulling this together is there?

***Reply****: The research significantly advances macroecology by addressing missing data through innovative imputation methods, revealing latent biases and gaps in biodiversity understanding. Unlike conventional trait datasets, the database aligns with fully-sampled phylogenies, enhancing its applicability. It incorporates spatial intersections, enabling mapping of various spatial patterns, facilitating research beyond drivers of richness and composition patterns. The database expands the taxonomic scale of comparative biology research in Tetrapoda, allowing quickly filtering of species according to biogeographic realm, major habitat, ecosystems. These features collectively offer a robust foundation for new avenues of research and insights in macroecology, demonstrating the potential to spur advancements in our understanding of the multiple aspects of tetrapod biodiversity.*

-	It would have been nice to make the code available for review.

***Reply****: The developed code is specifically designed for cloud computing on private servers and may not run on local machines. However, we provided an illustrative code designed with a data subset that is typically manageable on local machines (*[*https://zenodo.org/doi/10.5281/zenodo.10582069*](https://zenodo.org/doi/10.5281/zenodo.10582069)*).*

-	Also, line 670 says the database is available for reviewers at vertlife.org but I could not find it. I clicked on all the links under 'data' tab, but didn't see it anywhere. This was admittedly quite frustrating for me. I did download the supplementary file version and have a look, but the files there did not match up with the file names listed in 672-676.

***Reply****: At this stage, we are providing database files for review only as additional files. The TetrapodTraits and spatial intersections use taxonomical nomenclature directly imported from fully-sampled trees published for amphibians* [10]*, chelonians and crocodilians* [13]*, squamates and tuatara* [11]*, birds* [9]*, and mammals* [12]*.*

In conclusion, while I like the notion here presented by the authors, and I do see some value in the aggregation of these data, I think more convincing would be needed for (a) how much has been 'added' by the authors, (b) the potential uses, and (c) more clarity/structure and presentation of the database being presented.

***Reply****:* *Thank you very much for your supportive review, which provided rich details. In response to your valuable feedback, we have incorporated a new figure illustrating the novelty within TetrapodTraits. We also restructured the Methods sections and presented additional arguments to highlight the applicability of TetrapodTraits.*

**_________________________**

**Reviewer: 3**

Moura et al provide a new, compressive and phylogenetically imputed tetrapod trait database. They highlight groups and geographical regions where trait data is missing and where imputation of these values significantly alters underlying estimates for those traits. The work is clearly valuable and will provide a useful resource for many biogeographical studies in future.

***Reply****: Many thanks for your time and consideration in reviewing our manuscript. We truly appreciated it.*

I was surprised to see no discussion of the impacts of language on the collection of new data, or indeed on the estimation of gaps. Given the growing evidence of valuable ecological and conservation literature being locked away in non-English articles and journals (Amano et al, 2021), did the authors make any particular effort to source data from non-English sources? Are particular species traits more likely to be missed by this problem? The conclusion/discussion focussed instead on the latitudinal and trait-based natures of these gaps - might some of this be driven by our English lens?

***Reply****: While English predominates in the literature, our compilation aimed to include data from various sources, such as field guides and species descriptions published in Portuguese and Spanish. It is important to note that species-specific searches on platforms like Google Scholar often yield sources in languages beyond English, Spanish, and Portuguese. In such cases, we utilized digital translators to examine these sources. Language was not a barrier for data compilation, although we recognize that digitized PDFs of local journals were not always accessible or available for inspection. Additional details about the languages consulted during our searching procedure are provided in the 'Data Acquisition' subtopic within the Methods section.*

Given the comprehensive work the authors have gone to impute missing traits in this database using Rphylopars and missForest, i was surprised there was no presentation of estimates of the accuracy of these imputations. Could the authors not subsample/hide some traits and then impute them and estimate the accuracy of these processes?

***Reply****: We appreciate the reviewer's attention to the imputation process. However, we would like to highlight that we did conduct model validation, and the details can be found in the Supporting Information. Specifically, we employed a 10-fold cross-validation approach, subsampling all species with complete natural history data. We artificially relabelled their traits as missing data to assess the performance of the imputation models. We rebuild this cross-validation using the new analytical framework implemented in the revised manuscript (please see Figs S1-S3 in the supporting information).*

This is particularly true as the authors focus on the difference between "congruent or aggregated (as opposed to segregated)" gaps, but it is not clear how the accuracy of the imputation methods varies across this difference.

***Reply****: We apologize for any lack of clarity on this aspect. The performance of the imputation model is assessed at the species level, while the analysis of shared missingness is conducted at the genus or family level. This difference complicates direct comparisons between these aspects. However, we have included additional analyses demonstrating the lack of a relationship between trait biases and the number of imputed traits (Fig. S16). We have also illustrated the absence of an association between trait biases and the degree of shared missingness (Fig. S17) and have shown that trait biases are higher for species-poor samples, particularly under conditions of low data completeness (Figs. S18-S20).*

Line 321: 'binarized'

***Reply****:: done.*

Line 658: 'demonstrated' or 'we demonstrate'

***Reply****: done.*

Line 661: "Through careful imputation we uncover that these gaps have brought about attribute distributions that have strong geographical and taxonomic biases." Feels a bit convoluted.. perhaps "Through careful imputation we reveal that these gaps have caused strong geographical and taxonomic biases in trait distributions".

***Reply****: We rephrased the sentence as “Through careful imputation, we discovered that these gaps have led to attribute distributions with strong geographical and taxonomic biases”.*

#END OF REVIEW

_________________________________

**References we cited in this letter:**

1.	Deng Y, Lumley T. Multiple Imputation Through XGBoost. J Comput Graph Stat. 2023; 1–19. doi:10.1080/10618600.2023.2252501

2.	Chen T, Guestrin C. XGBoost: A scalable tree boosting system. Proceedings of the 22nd ACM SIGKDD International Conference on Knowledge Discovery and Data Mining. New York, NY, USA: ACM; 2016. pp. 785–794. doi:10.1145/2939672.2939785

3.	Little RJA. Missing-Data Adjustments in Large Surveys. J Bus Econ Stat. 1988;6: 287. doi:10.2307/1391878

4.	Nakagawa S, Freckleton RP. Model averaging, missing data and multiple imputation: a case study for behavioural ecology. Behav Ecol Sociobiol. 2011;65: 103–116. doi:10.1007/s00265-010-1044-7

5.	Penone C, Davidson AD, Shoemaker KT, Di Marco M, Rondinini C, Brooks TM, et al. Imputation of missing data in life-history trait datasets: which approach performs the best? Methods Ecol Evol. 2014;5: 961–970. doi:10.1111/2041-210X.12232

6.	Taugourdeau S, Villerd J, Plantureux S, Huguenin‐Elie O, Amiaud B. Filling the gap in functional trait databases: use of ecological hypotheses to replace missing data. Ecol Evol. 2014;4: 944–958. doi:10.1002/ece3.989

7.	IUCN - International Union for Conservation of Nature. IUCN Red List of Threatened Species. In: Version 2018.2. 2018 p. www.iucnredlist.org.

8.	Roll U, Feldman A, Novosolov M, Allison A, Bauer AM, Bernard R, et al. The global distribution of tetrapods reveals a need for targeted reptile conservation. Nat Ecol Evol. 2017;1: 1677–1682. doi:10.1038/s41559-017-0332-2

9.	Jetz W, Thomas GH, Joy JB, Hartmann K, Mooers AO. The global diversity of birds in space and time. Nature. 2012;491: 444–448. doi:10.1038/nature11631

10.	Jetz W, Pyron RA. The interplay of past diversification and evolutionary isolation with present imperilment across the amphibian tree of life. Nat Ecol Evol. 2018;2: 850–858. doi:10.1038/s41559-018-0515-5

11.	Tonini JFR, Beard KH, Ferreira RB, Jetz W, Pyron RA. Fully-sampled phylogenies of squamates reveal evolutionary patterns in threat status. Biol Conserv. 2016;204: 23–31. doi:10.1016/j.biocon.2016.03.039

12.	Upham NS, Esselstyn JA, Jetz W. Inferring the mammal tree: Species-level sets of phylogenies for questions in ecology, evolution, and conservation. PLOS Biol. 2019;17: e3000494. doi:10.1371/journal.pbio.3000494

13.	Colston TJ, Kulkarni P, Jetz W, Pyron RA. Phylogenetic and spatial distribution of evolutionary diversification, isolation, and threat in turtles and crocodilians (non-avian archosauromorphs). BMC Evol Biol. 2020;20: 81. doi:10.1186/s12862-020-01642-3
