# Supplementary material for: A phylogeny-informed characterisation of global tetrapod traits addresses data gaps and biases
Source: PLoS Biol. 2024 Jul 11;22(7):e3002658. doi: 10.1371/journal.pbio.3002658 (PMC11239118; doi:10.1371/journal.pbio.3002658)
Supplement: Attachment — Submitted filename: RebuttalLetter_2nd.docx [file pbio.3002658.s026.docx]

April 19, 2024

Dear Dr. Roberts,

Thank you for the letter dated March 21 in which you invite us to submit a revised version of the manuscript #PBIOLOGY-D-24-00327R1 “*A phylogeny-informed characterisation of global tetrapod traits addresses data gaps and biases*”.

We appreciate the constructive feedback provided during the PBiol review process, which has helped us improve the quality of our research. In the latest decision letter on our manuscript, two requirements were made, as transcribed in the following.

*…We discussed the reviews and the cross-comments with the Academic Editor, who felt that on balance your Resource would be a useful contribution, and that we should invite a further revision. However, we will need* ***full provision of the code and data****, and the Academic Editor emphasised that* ***Figure should be made a "more visually attractive and readable****..."*

Based on the requirements above, we unpacked our answer in two points addressed in the revised manuscript. All line numbers refer to the revised manuscript, without tracked changes:

*1.	“full provision of the code and data”*

Reply: We agree with the handling editor and reviewers that the code and data need to be fully available. Moreover, not only do we support this view, but we also emphasize that the code needs to be reproducible by third parties, including individuals with limited or no access to private cloud computing services. That’s the major reason why we have prepared a highly detailed and commented code, rarely found in the literature, to reproduce the analysis of our manuscript using local machines. This code is hosted in ZENODO, as informed in the manuscript ([10.5281/zenodo.10582069](https://zenodo.org/records/10582070)). Perhaps, the reviewer missed that information. The provided code reproduces all figures of the manuscript, including those reported in the main text and supplementary material. All table and spatial file (e.g., shapefiles) needed to prepare those figures are also provided. To allow code execution in local machines, with somewhat limited hardware relative to private clusters, we provide an illustrative data subset with 1K mammal species. We believe this approach promotes inclusivity in science while keeping reproducibility and transparency.

The full TetrapodTraits database, along with the 10K tables containing imputed values, as well as the spatial intersections across the 110 × 110 km grid cell scheme used for our mapped presentations, are available at [10.5281/zenodo.10530618](https://doi.org/10.5281/zenodo.10530618).

*2.	“Figure should be made a more visually attractive and readable”.*

Reply: We are unsure whether the handling editor and/or reviewers had access to low-resolution files. In this revised version, all manuscript figures are provided in vectorized format (PDF) outside the MS (not embedded in the text) to facilitate easy reading at any desired zoom level.

We would like to highlight that our goal for figure presentation was to convey information on multiple aspects of TetrapodTraits while minimizing the number of graphical pieces. Most figures, therefore, include more than one “layer” of information, being accompanied by inset plots illustrating additional data properties and structure. Particularly for the maps provided, while clear latitudinal/geographical patterns can be found regarding the magnitude (Fig. 3) and direction (Fig. 6) of missing data, we add brief histograms, donut plots, and latitudinal plots to instantaneously inform the audience on evident data patterns.

Color schemes and color ramps were selected to be colorblind safe and print-friendly, using well-known strategies of color visualization. We were careful to use the separate color scheme to communicate the magnitude (*viridis* color ramp) and direction (*diverging* color ramp) of data missingness to avoid conflicting interpretation of distinctive data patterns. Since our manuscript covers tetrapods, we also built all major graphical pieces separately for each major taxonomic group (amphibians, squamates, turtles and crocodilians, birds, and mammals), all provided as supplementary material to reach an audience potentially interested in outputs for specific taxonomic groups.

\### REVIEWER #1

I congratulate the authors in their attempts to correctly account for missing data using multiple imputation. This was my main criticism in my previous review, and it looks like the authors have done a great job. Providing 10000 multiply-imputed datasets is a great way of allowing researchers to deal with imputation issues, without having to do the imputations themselves, which means the authors have control of the imputation process, and hopefully have done a better job than most researchers could do (due to lack of experience with missing data). In addition, the authors have identified which cells in the data table have been imputed so in an extreme case, researchers could do their own imputation. This is the best of both worlds. I need to point out that the use of predictive mean matching (PMM) for multiple imputation is not a phylogenetically informed procedure unless the probability of accepting a value is proportional to the phylogenetic distance between the imputed and donor species, resulting in a phylogenetic bias towards values from near phylogenetic relatives. This is probably a minor point but the authors should recognise this. I still disagree with the use of NRMSE and PFC. These measures of imputation quality are unnecessary in a multiple-imputation framework as during the imputation we want to increase variability into our imputations. In a multiple imputation framework, we don't want NRMSE and PFC to be too low because we are not trying to replace our missing values with the "true" value (which remains unknown), merely drawing from the approximate posterior distribution of each missing value. So in a multiple-imputation framework, these statistics are unnecessary because they don't measure anything meaningful about the quality of the imputations. A far better approach, which is implemented in the mice package, is to look at the densities of the imputed values compared to the true values, for each variable. We do want the distributions of the imputations to have a similar shape and location to that of the observed values, as departures from this means that our imputations are not enough like our non-missing data to be values that "could" have occurred. This can be a graphical check, and you wouldn't need to look at all 10000 imputations. A random sample of 10 - 20 would suffice. Although the imputations here were not produced by mice, it is a comparatively simple procedure to form a mice-compatible data set and use the densityplot() function in mice. See chapter 6 in Stef van Buuren's book on mice, which is available online: https://stefvanbuuren.name/fimd/ Otherwise, the paper is a good contribution and will form the basis for many new studies.

Reply: Thank you for dedicating your time to reviewing our manuscript. We followed the reviewer’s suggestions and compared the distribution of predicted and observed values for each target variable in our testing dataset. For continuous variables (body length and body mass), we present histogram depicting the distribution of observed and predicted values, whereas for binary variables, we show barplots indicating the frequency of 0-1 values across observed and predicted data entries. We highlight that shape and location (continuous variables) and frequency (binary variables) are remarkably similar between observed and predicted data entries. Please, see Fig. S4 in Supporting Information.

*PMM* assigns imputed values to each missing entry based on a group of k donors whose predicted values are the most similar among the observed entries. The donor’s predicted value is influenced by its placement in the multivariate predictor space, which includes several phylogenetic filters and natural history traits (body length, body mass, microhabitat, and activity time). Therefore, our utilization of *PMM* is guided by the phylogenetic relationships among species. However, in a rare event in which a given *XGBoost* model drops all phylogenetic filters during the “tree growth” phase, the imputed values would not take the phylogeny into account. We have included a brief sentence in the Methods section to address this (line 402-405, transcribed below).

*“The selection of donors, crucial for predictive mean matching in mixgb models, is based on exploring the multivariate predictor space (i.e, phylogenetic filters and natural history traits) during the tree building process in XGBoost models.”*

We used *NRMSE* and *PFC* to tune hyperparameters in *XGBoost* models and assess the initial predictive accuracy of the trained models themselves. Our aim was to ensure high in-sample performance before moving to predict/impute missing data entries. Once the models were trained, our imputation framework considered four distinct sources of variability. We added a new paragraph in the ‘Phylogenetic Multiple Imputation’ section to highlight this (lines 427-433, transcribed below):

*“In our approach, there are four sources of variability when producing multiple imputed values for each missing data entry. Firstly, the PMM with 10 donors per entry increases variability by avoiding a reduced number of donors. Secondly, the replication of the PMM technique 10 times to potentially select multiple values. Thirdly, the 10-fold cross-validation trained 10 different mixgb models per target variable. Finally, we incorporated 100 fully-sampled phylogenetic trees, enabling species with imputed evolutionary relationships to vary their position in the multivariate predictor space, guided by phylogenetic filters.”*

\### REVIEWER #2

This is a re-review of the manuscript titled "A phylogeny-informed characterization of global tetrapod traits addresses data gaps and biases".

Reply: Many thanks for your time and consideration in reviewing our manuscript. We truly appreciated it.

Overall, I appreciated the time the authors spent in revising the manuscript. However, at this point, I still would say I am unconvinced that this paper represents any kind of novel approach or advance or has significant ability to spur on future research. I actually found the responses to the comments by the Reviewers to be quite superficial, sometimes not really addressing the meat of the question/comment. For example, all because the data are aligned with phylogenies does not mean it represents any kind of advance - this is relatively easy to do with a couple of lines of code to align data with phylogenies.

Reply: We respectfully disagree with reviewer #2's assertion that our modifications were superficial. The statistical framework has been completely rebuilt, with every analysis and figure remade, along with significant restructuring of the text flow and accompanying data files. The overall results represent the synthesis of over 120,000 output files totaling nearly 600GB, with the code provided covering 6K lines, commented through more than 25K words. The perception of our changes as ‘superficial’ could potentially be explained by the robustness of our findings between the first and second manuscript versions. Even after completely changing the statistical framework – transitioning from single imputation (using rphylopars and missforest) to a multiple imputation approach (using xgboost and ppm) – our results remained consistent.

Speaking of code, if I interpret correctly, the code used is only available in a small subset? AND the data are not currently available for review? I apologize if I'm misinterpreting, but in theory if a reviewer asks for where the data is and that they couldn't find it (even though it is implied it is available at vertlife.org) and then there isn't really a legitimate response, I find this frustrating as a reviewer. Moreover, I suggest the authors have a read of this: https://journals.plos.org/plosbiology/article?id=10.1371/journal.pbio.3002516.

Reply: We have prepared a highly detailed and commented code rarely found in the literature, which reproduces the analysis and figures of our manuscript using local machines ([10.5281/zenodo.10582069](https://zenodo.org/records/10582070)). In our previous resubmission, we provided the full database as zip-file for review purposes only, but we are unsure if the reviewer #2 missed those files or did not have access to them. The complete database is now published at [10.5281/zenodo.10530618](https://doi.org/10.5281/zenodo.10530618).

I did appreciate Figure 1, but this confirms my original point that not all that much is being added here. Plus, the 'other sources' is still vague! "often compiled from field guides and taxonomic-specific literature for tetrapod species". I honestly don't know how to interpret that, and in the original review, it seems quite specific what the question was. In theory, this should be quite easy to quantify and respond to the original reviewer query about.

Reply: Each data entry in TetrapodTraits database is accompanied by fields informing specific sources (e.g., SourceBodyLength, SourceBodyMass, SourceActTime, SourceHabitat, SourceMajorHabitat, SourceEcosystem, and SourceInsularity). These sources include published works such as articles, books, book chapters, and datapapers, as well as grey literature such as technical reports, government documents, and dissertations. All data entries derived from sources other than “datapaper” represent novel compilation efforts or unpublished data, in addition to imputation themselves. This novel/revised information comprehends about one third of all data entries on natural history traits informed in our database. We have revised and classified all sources into major reference types (datapapers, articles, books, grey literature, unpublished data, and websites) and rebuilt Figure 1 using a different perspective focused on source type instead of source ID.

And then, there still remains the philosophical question of why simply using off-the-shelf imputation methods warrants publication. I would lean towards it doesn't. I do appreciate, however, that the authors did more work on using more reliable imputation methods and making the data available for all imputations. But, returning to the point above, this is relatively simple and if ALL code and a workspace where this was done is not provided, then I simply cannot judge it meaningfully, and therefore cannot recommend publication of this manuscript in PLoS Biology.

Reply: We acknowledge Reviewer #1's specific support for the current version, noting their statement: “I congratulate the authors on their attempts to correctly account for missing data using multiple imputation. This was my main criticism in my previous review, and it looks like the authors have done a great job.” This positive feedback underscores our efforts to address critiques and improve the manuscript. Regarding the availability of our code, we emphasize that it has been made runnable on local machines to enhance inclusivity, transparency, and reproducibility in science.

END OF REVIEW

############

Mario R. Moura, on behalf of the authors.

Visiting Professor, Department of Animal Biology, Universidade Estadual de Campinas, Brazil
